# Phylogeography of *Diptychus maculatus* (Cyprinidae) endemic to the northern margin of the QTP and Tien Shan region

**DOI:** 10.1186/s12862-016-0756-3

**Published:** 2016-09-09

**Authors:** Guogang Li, Yongtao Tang, Renyi Zhang, Kai Zhao

**Affiliations:** 1 Key Laboratory of Adaptation and Evolution of Plateau Biota, Northwest Institute of Plateau Biology, Chinese Academy of Sciences, No. 23 Xinning Road, Xining, Qinghai 810001 China; 2 Laboratory of Plateau Fish Evolutionary and Functional Genomics, Northwest Institute of Plateau Biology, Chinese Academy of Sciences, No. 23 Xinning Road, Xining, Qinghai 810001 China; 3 University of Chinese Academy of Sciences, Beijing, China

**Keywords:** Phylogeography, Mitochondrial and nuclear markers, *Diptychus maculatus*, Tienshan Mountains, Qinghai-Tibetan Plateau

## Abstract

**Background:**

Phylogeography and historical demography of the cyprinid fish *Diptychus maculatus* (subfamily Schizothoracinae) are evaluated across three river systems in the Northern Qinghai-Tibetan Plateau (QTP) and Tien Shan range: the Indus River, Tarim River and Ili River.

**Results:**

Results from both mtDNA (16S rRNA, Cyt *b* and D-loop) and nucDNA (RAG-2) resolved four reciprocally monophyletic clades, representing populations from Indus River, South Tarim River, North Tarim River and Ili River, respectively. The divergence times was estimated to be 1.5–2.5 Mya. It is consistent with the hypothesis that the split of four clades is the consequence of vicariance resulting from both the intensive uplift of QTP and Tien Shan as well as the resultant expansion of the Taklimakan Desert. Several lines of evidences indicate dynamic demographic histories for the populations, with late Pleistocene and Holocene population bottlenecks and expansions except the Indus River.

**Conclusions:**

Our results clearly depicted the phylogenetic relationship of *D. maculatus* from Indus River, Tarim River and Ili River. The analyses implicated the relationship among the distribution of *D. maculatus*, paleo-drainages and geographic events, and implied the existence of the South Tarim River in history.

**Electronic supplementary material:**

The online version of this article (doi:10.1186/s12862-016-0756-3) contains supplementary material, which is available to authorized users.

## Background

Tibetan Movement was the most important geological event in the Quaternary period, causing a series of large geomorphological adjustments and producing the present geomorphic, hydrologic as well as tectonic configurations [1]. According to Li [1], this significant movement included A, B and C phases occurring at 3.6, 2.5 and 1.7 Ma respectively. The Phase C marked the beginning of a new era of “World Ridge” development [2]. Especially between 1.1 and 0.6 Ma, the so-called the Kunlun-Yellow River Movement elevated Qinghai-Tibetan Plateau (QTP) rapidly to an average height of 3000 m with mountains up to over than 4000 m [3, 4]. Followed by Tibetan Movement, the Tien Shan area elevated again and reached the current height and formed the modern geomorphologic pattern of the northwestern China [5]. Meanwhile, the QTP blocked the sea winds of the Indian Ocean from going further into northern China, causing the development of the eastern monsoon and the formation of an arid and semi-arid climate in this area. The direct outcome of the dryness was the development and spread of Taklimakan Desert, the second largest shifting sand desert in the world, which covered the Tarim Basin surrounded by QTP and Tien Shan [2, 6, 7]. The expansion of Taklimakan Desert led to the change of watercourses, which deteriorated the ecosystem in the area [8].

The theories of modern phylogeography are based on the assumption that the biological evolution and geographical changes are synchronized [9]. Since the geographic changes will result in spatial segregation [10], characterizing the current distribution of the endemic fauna of on QTP may help us to explore the geological histories of these mountain-river systems within and around the region. It is well accepted that the evolution and distribution patterns of native freshwater fishes reflect the paleogeographical complexity of rivers [11–13]. The evolution and distribution patterns of schizothoracine cyprinids reflects the paleogeographical history of the QTP and adjacent regions [14], especially the evolution of paleo-drainage systems, of the area [15].

**Fig. 1:**
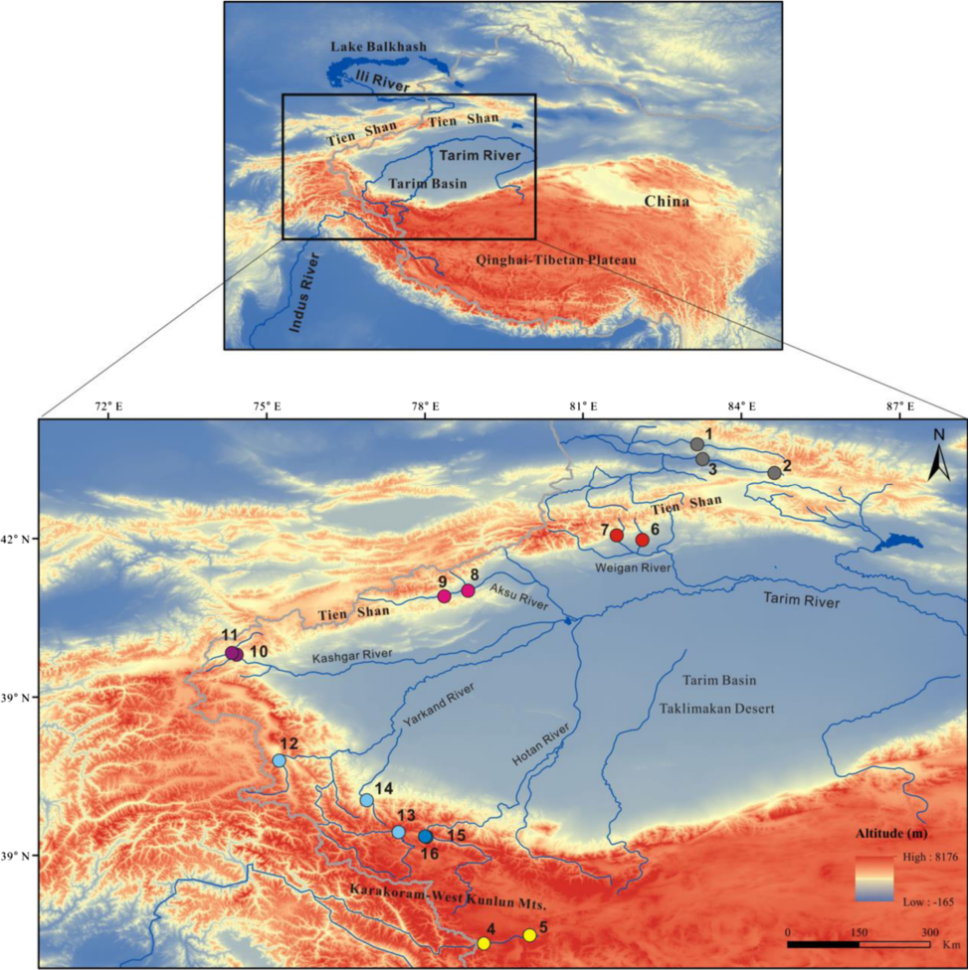
Geographic location of *Diptychus maculatus* in the Tien Shan and Qinghai-Tibetan Plateau. Locality codes correspond with those in Table 1

*Diptychus maculatus* is the species in a monotypical genus of the subfamily Schizothoracinae (Cyprinidae), which is well adapted to high-mountain streams with low temperature and hypoxia on QTP and Tien Shan [16, 17]. It is mainly distributed in the Indus River system, the Tarim River Basin and the Ili River-Balkhash Lake Basin. These big river systems were originated from the surrounding mountains. The Tarim Basin, the largest endorheic basin on our planet (Fig. 1), is bordered by the Tien Shan Mountain in the north and by Karakoram-West Kunlun Mountain in the south, which is also at the north edge of the QTP. From the north slope of Tien Shan, the Ili River flows northward into the Lake Balkhash. The Indus River flows southward to the Indian Ocean, which was originated in the Karakoram Mountain. The current Tarim River system has a five-source-one-mainstream pattern of which all tributaries originate in the south slope of Tien Shan and the north slope of West Kunlun Mountain. They flow into the Tarim Basin, across the desert from west to east, and converge on in the final destination in Lop Nur. Due to the uplift of QTP and Tien Shan in Quaternary period, the geographical and natural environments varied dramatically in the three river basins, especially in Tarim River, which is quite different from its paleo-pattern (Additional file 1: Figure S1). *D. maculatus*, as the endemic fishes, experienced all the environmental and geographic changes, hence, we hypothesize that the present distribution of *D. maculatus* is the consequence of a series of geomorphological adjustments in the region.

The previous researches on the *D. maculatus* focused on two aspects. One was the correlation between phenotypic variation (scale and grill rake numbers) and river systems, implying the species were adapted to the local aquatic environments by morphological changes [16, 18]. The other is the discovery of phylogeographic structure of this species [19, 20]. However, these studies were largely questioned and unable to uncover the entire evolutionary relationship between the fish and the mountain-river systems because of the inadequate samples size, incomplete geographic coverage (for example, no Indus River), and only mtDNA, the matrilineal marker, were used [15, 21–23]. To overcome these issues, we reconstructed the phylogeographic histoy of *D. maculatus* by applying both mtDNA and nucDNA markers together with the intensive sampling covering all the habitats. In the current study, we successfully describe the phylogeography and evolutionary history of the species. Meanwhile, our analyses supported the association among the uplift of QTP and Tien Shan, the paleodrainage systems as well as the present distribution of *D. maculatus*.

## Results

### Sequence data

Three mtDNA and one nucDNA markers of *D. maculatus* were sequenced: the aligned 16S rRNA sequences (1118 bp) with 47 variable sites and 40 parsimony-informative sites; the Cytochrome *b* (Cyt *b*) sequence (total length of 1140 bp) with 135 variable sites and 127 parsimony-informative sites; the D-loop sequences (708 bp) with 63 variable sites and 61 parsimony-informative and two insertion/deletion sites; the segment from RAG-2 (1250 bp) with six variable sites.

We detected seven RAG-2 haplotypes from all 261 samples of *D. maculate*. Among the combined mitochondrial sequences (2966 bp) from all individuals, 70 unique haplotypes in total were identified (Table 1 and Additional file 2: Table S1). The mean divergence among mtDNA haplotypes was 2.19 %.

**Table 1:** The sampling locations, number of specimens (N), and river systems for the samples of *Diptychus maculatus* used in this study. The location codes correspond to those in Fig. 1

Clade/subclade	Location code	Coordinate	Altitude (m)	Sampling location	N
Ili River	1	83°09′45.10″E /43°47′52.65″N	1465	Kashi River, Nilka	16
Ili River	2	84°37′32.04″E/43°15′16.21″N	2028	Kunes River, Kunes	23
Ili River	3	83°15′49.60″E/43°31′10.00″N	981	Kunes River, Musi	8
Indus River	4	79°07′00.64″E/34°19′57.10″N	4810	Chang-chenmo River, Rutog	24
Indus River	5	79°59′26.02″E/34°29′14.12″N	5164	Qiangchenmo River, Rutog	10
North Tarim River/Weigan River	6	82°07′27.64″E/41°58′29.79″N	1514	Karasu River, Baicheng	45
North Tarim River/weigan River	7	81°38′21.17″E/42°03′44.28″N	1818	Taileweiqiuke River, Baicheng	43
North Tarim River/Aksu River	8	78°48′56.09″E/41°00′51.11″N	1747	Aksu River, Akqi	7
North Tarim River/Aksu River	9	78°21′45.60″E/40°54′31.82″N	2029	Aksu River, Akqi	16
North Tarim River/Kashgar River	10	74°25′53.67″E/39°48′09.72″N	2452	Kezi River, Wuqia	1
North Tarim River/Kashgar River	11	74°20′29.91″E/39°50′07.43″N	2490	Kezi River, Wuqia	3
South Tarim River/Yarkand River	12	75°14′07.59″E/37°47′33.90″N	3067	Tashkurgan River, Tashkurgan	6
South Tarim River/Yarkand River	13	77°29′54.90″E/36°26′35.50″N	4002	Yarkand River, Yecheng	34
South Tarim River/Yarkand River	14	76°53′49.20″E/37°02′52.30″N	2459	Tizinapu River, Yecheng	17
South Tarim River/Hotan River	15	78°01′42.60″E/36°21′02.40″N	3669	Karakash River, Pishan	4
South Tarim River/Hotan River	16	77°59′56.10″E/36°21′40.00″N	3642	Karakash River, Pishan	4

### Phylogenetic analyses

**Fig. 2:**
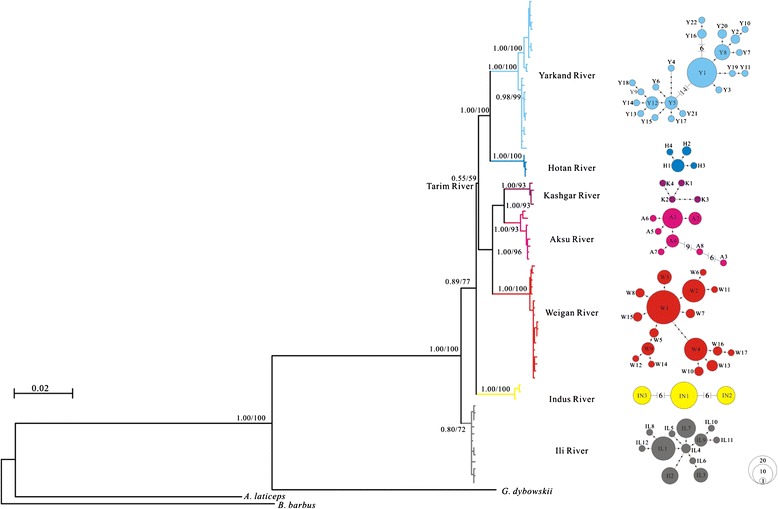
The Bayesian inference tree for *Diptychus maculatus* based on the 70 haplotypes from combined mtDNA. Numbers on branches indicate, posterior probability in BI analyses followed by bootstrap supports for ML the node. The corresponding median-joining network based on the combined sequence data for each clade is depicted to the right of each clade. The haplotype numbers correspond to those in the Additional file 2: Table S1. The circle sizes represent the approximate numbers of individuals, and the scale is provided in the lower right corner. The black dots indicate the nucleotide substitutions inferred for that branch. The geographical origins of the haplotypes are illustrated by the same colors used Fig. 1

Based on the combined mtDNA data, the same topologies were recovered by both the Bayesian and ML trees (Fig. 2). The result showed a phylogeographic structure in which four distinct haplogroups corresponded well to four independent evolutionary clades, representing populations from South Tarim River, North Tarim River, Indus River and Ili River (Figs. 1 and 2). The Taklimakan Desert separates the South and North Tarim River; while the Ili River and the North Tarim River are separated by Tien Shan; as the northwest edge of QTP, Karakoram Mountain separates the Indus River and the South Tarim River (Figs. 1 and 2). Within North Tarim River clade, three subdivisions (Kashgar, Aksu and Weigan River)were further classified, in a good agreement with three main tributaries of the North Tarim River (Fig. 2; Additional file 2: Table S1). Within South Tarim River clade, two subdivisions (Yarkand and Hotan River) were corresponded with two tributaries (Fig. 2; Additional file 2: Table S1). Moreover, the haplotype network for the Yarkand subclade contained two clusters of haplogroups that were separated by 14 mutations (Fig. 2). No haplotypes were shared between clades or subclades.

**Fig. 3:**
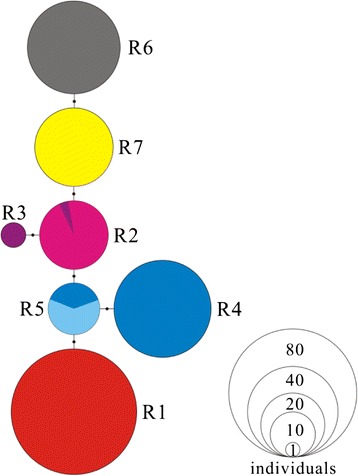
Median-joining network based on the RAG-2 sequence data for all individuals of *Diptychus maculatus*. The haplotype numbers correspond to those in the Additional file 2: Table S1. The circle sizes represent the approximate numbers of individuals, and the scale is provided in the lower right corner. The black dots indicate the nucleotide substitutions inferred for that branch. The geographical origins of the haplotypes are illustrated by the same colors used Fig. 1

The haplotype network of the nucDNA marker, RAG-2 was inconsistent with the phylogenetic results of the mtDNA data (Figs. 2 and 3). The Ili River collection (*N* = 47) and Indus River collection (*N* = 34) were monomorphic for haplotypes R6 and R7 respectively. Also, within the North Tarim River, haplotype R1 was monomorphic in the Weigan River collection (*N* = 34). In the Kashgar River collection (*N* = 4), three of them were monomorphic for haplotype R3, and the remaining one shared haplotype R2 with the Aksu River collection (*N* = 23). Additionally, within the South Tarim River and the Yarkand River collection (*N* = 57), 52 samples were monomorphic for haplotype R4, and the remaining five shared the same haplotype R5 with the Hotan River collection (*N* = 8) both (Fig. 3; Additional file 2: Table S1). The nucDNA marker indicated that Weigan River population (haplotype R1) was genetically closer to South Tarim population (haplotype R5) with only one mutation than to North Tarim population (haplotype R2) with at least three mutations, which was quite different from the mtDNA analysis.

### Nucleotide diversity and genetic structure

The total haplotype (*h*) and nucleotide (π) diversities were significantly high for all the four clades (Table 2). However, the nucleotide diversity was very different for among clades, higher in the South Tarim clade (0.6996 % ± 0.3469) and North Tarim clade (0.9799 % ± 0.4779) than quite low in the Ili River clade (π = 0.0806 % ± 0.0495) and Indus River clade (π = 0.0943 % ± 0.0568) (Table 2).

**Table 2:** Genetic diversity and neutrality test results for clades/subclades of *Diptychus maculatus*

Clade/Subclade	Gene diversity	Nucleotide diversity (%)	Tajima’s D (*P* value)	Fu’s *F*s (*P* value)
Ili River	0.8474 ± 0.0305	0.0806 ± 0.0495	−0.3594 (0.4178)	−2.9242 (0.0972)
Indus River	0.6275 ± 0.0537	0.0943 ± 0.0568	2.5050 (0.9956)	5.8652 (0.9834)
North Tarim River	0.9014 ± 0.0164	0.9799 ± 0.4779	0.8319 (0.8464)	10.2111 (0.9606)
South Tarim River	0.8712 ± 0.0358	0.6996 ± 0.3469	0.1995 (0.6618)	1.9915 (0.7600)
Weigan River	0.8461 ± 0.0252	0.0921 ± 0.0547	−0.6772 (0.2736)	−4.5150 (0.0580)
Aksu River	0.7747 ± 0.0713	0.0906 ± 0.0558	−1.6347 (0.0404)	−0.8623 (0.3454)
Kashgar River	1.0000 ± 0.1768	0.1349 ± 0.1014	−0.8173 (0.1418)	−0.8247 (0.1426)
Yarkand River	0.8365 ± 0.0448	0.2623 ± 0.1375	−0.0126 (0.5646)	−2.5482 (0.2250)
Hotan River	0.7500 ± 0.1391	0.0313 ± 0.0273	−0.8125 (0.2688)	−1.3872 (0.0446)
All samples	0.9620 ± 0.0042	2.1093 ± 1.0101	1.7501 (0.9636)	14.0746 (0.9438)

To clarify the phylogenetic structure, analyses of molecular variance (AMOVA) was conducted based on the combined mtDNA data. The result showed that four clades accounted for most of variation (65.77 %), rather than three clades (39.51 %) (Table 3). Distributed respectively among the groups of the North Tarim River and the South Tarim River, 96.28 and 87.92 % of total variation were observed, with only 0.04 and 4.85 % of the variation among populations within the groups (Table 3).

**Table 3:** Summary of results of the hierarchical analysis of molecular variance (AMOVA) for *Diptychus maculatus*, All *P* < 0.001

Grouping option	% Among groups	% Among populations within groups	% Within populations	Φ_CT_	Φ_ST_	Φ_SC_
Three groups	39.51	56.90	3.60	0.3951	0.9640	0.9405
Four groups	65.77	30.46	3.77	0.6577	0.9623	0.8899
Seven groups	94.63	1.48	3.88	0.9463	0.9612	0.2765
Within north Tarim River	96.28	0.04	3.68	0.9628	0.9632	0.0120
Within south Tarim River	87.92	4.85	7.24	0.8792	0.9277	0.4012

As suggested by the phylogenetic inferences (Fig. 2), the highest and most significant values of pairwise comparisons of the genetic differentiation (*F*_ST_) were detected between all the four clades averaging more than 0.962, also between all the subclades averaging more than 0.940 (Table 4 and Additional file 3: Table S2). The values of *F*_ST_ within the clades were high and significant except in the Indus River clades. In contrast, the values of *F*_ST_ within the subclades were very low and not significant except that within Yarkand River. Mean divergence values between populations also strongly supported four clades inferences (Additional file 4: Table S3).

**Table 4:** Summary of average pairwise *F*_ST_ values for *Diptychus maculatus* within and among geographical zones and subdivisions

	*F*_ST_		*F*_ST_
Within Ili River clade	0.442	Between Weigan and Aksu	0.964
Within Indus River clade	−0.069	Between Weigan and Kashgar	0.962
Within North Tarim River clade	0.768	Between Aksu and Kashgar	0.940
Within South Tarim River clade	0.708	Within Weigan River subclade	0.017
Between Ili and Indus	0.966	Within Aksu River subclade	−0.051
Between Ili and North Tarim	0.970	Within Kashgar River subclade	0.090
Between Ili and South Tarim	0.966	Between Yarkand and Hotan	0.953
Between Indus and North Tarim	0.968	Within Yarkand River subclade	0.521
Between Indus and South Tarim	0.962	Within Hotan River subclade	−0.206
Between North and South Tarim	0.966		

### Demographic history

The estimated time to the most recent common ancestor (TMRCA) with 95 % highest posterior density (HPD) was showed in Additional file 5: Figure S2. The TMRCA of the in-group was estimated around 2.07 Ma with a 95 % HPD range from 1.71 to 2.45, indicated Early Pliocene divergences. The separation of Indus River and Tarim River clades occurred at 1.87 Ma with a 95 % HPD ranging from 1.55 to 2.19. The divergence time of North and South Tarim River clades by Taklimakan Desert was 1.77 Ma with a 95 % HPD ranging from 1.47 to 2.09.

**Fig. 4:**
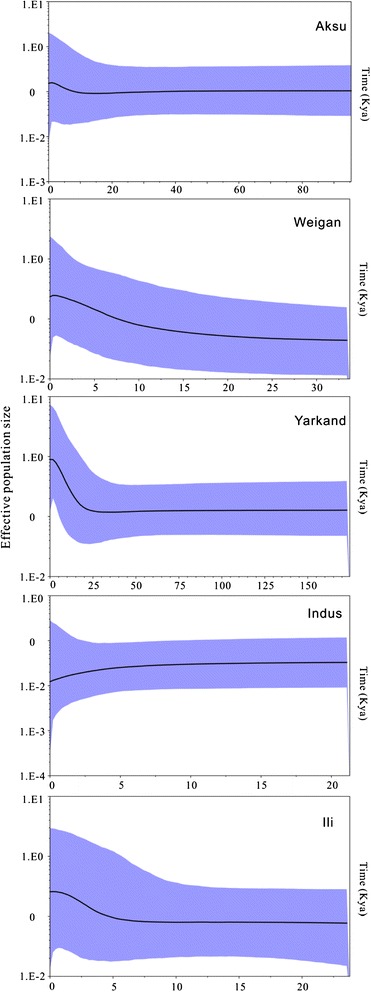
Bayesian skyline plots (BSPs) of five allopatric clades/subclades of *Diptychus maculatus*. The x-axis represents time in units of 1000 years. The y-axis represents effective population size as *N*_*e*_τ on a log scale. The black line depicts the median population size, and the shaded areas represent the 95 % highest posterior density intervals

Historical demography for the clades Tarim, Ili and Indus River provided the independent evolutionary process of *D. maculatus* from different drainages (Fig. 4; Table 2). The Tajima’s D values and Fu’s *F*s values indicated the population experienced a quick expansion in all clades and subclades except in the Indus River clade. Correspondingly, the star-like structure of haplotype networks within the eight clades and subclades also suggested the expansion event in the Ili River as well as North and South Tarim rivers (Fig. 2).

The Bayesian skyline plots (BSPs) showed that the population size was not statistically significant changed because of broadly overlapping HPDs (Fig. 4). However, the trend for each population size was consistent with the neutrality test results. For the North Tarim River clade (Weigan and Aksu River), BSP indicated a moderate and recent (25 and 10 Kya) expansion, whereas BSP of South Tarim River clade (Yarkand River) indicated a strong expansion (of about 25 Kya) (Fig. 4). BSP of Ili River clade was stable until rapidly expanded about 7–5 Kya. BSP demonstrated that the Indus River population kept shrunk and dramatically reduced at about 5 Kya. Finally, BSPs indicated the population of all the clades were depressed about 1 Kya.

## Discussion

### Phylogeography and historical demography of *D. maculatus*

The current study drew a whole picture of phylogeography of *D. maculatus* for the first time by the intensive sampling across the north of QTP and adjacent regions, from three major drainages: Indus River, Tarim River and Ili River. We identified four clades, of which two corresponded geographically to the Indus River and Ili River respectively, and the other two from Tarim River population separated by the Taklimakan Desert (Figs. 1 and 2).

The divergence history of *D. maculatus* clearly showed that the split of the species occurred when the geographic events happened. For example, the estimated producing times of four clades corresponded to the timing of the uplift of Tien Shan in the Early Pleistocene period and the third phase of the “Tibetan movement” at 1.7 Ma [1]. The forming of three subclades within the North Tarim River also occurred when Tien Shan uplifted in the Early Pleistocene period. The two subclades within the South Tarim River clade were split during Kunlun-Yellow River Tectonic Movement (1.1–0.6 Mya) [1]. Therefore, we concluded that the allopatric divergence producing four clades and five subclades of *D. maculatus* was likely associated with the vicariance caused by the rapid uplift of Tien Shan and QTP.

Several lines of evidence confirmed a dynamic demographic history for the five clades/subclades resolved in *D. maculatus*. First, star-like haplotype networks with high level of haplotype diversity and low nucleotide diversity indicated all population but Indus River clade experienced the bottleneck effect followed by the population expansion [22, 24]. Second, the negative Tajima’s D and Fu’s *F*s statistics together with the BSPs indicated Yarkand, Aksu, Weigan and Ili river populations expanded in a late Pleistocene or Holocene.

The principal reason of the demographic changes was the climatic and environmental alterations caused by the geographic events. In the Late Pleistocene and Holocene era, the large-scale and rapid uplift of QTP resulted in the dramatic changes in both climate and ecology [2]. The BSPs for Yarkand and Weigan Rivers indicated that the expansion began 25 Kya, and the expansion for Aksu River began 10 Kya, coinciding with the uplift of Karakoram-West Kunlun Mts. This movement allowed the westerly wind to come into northern slope of Karakoram-West Kunlun Mts., which sequentially lead to the warm-humid climate and river development in the area [25]. In contrast, because the Indus River population was located in the southern slope of Karakoram-West Kunlun Mts. where the westerly wind could not reach, the population size was gradually declined as indicated by the BSP analysis. Notably, samples of Indus River population were collected from very high altitude (4810 m), and the harsh conditions presumably had influences on the genetic variations of the population. The small number of haplotypes discovered in Indus River population resulted in the lack of signal to evaluate the dynamic demographic history. The Ili River, protected by the Tien Shan from the Taklimakan Desert, was consistently under the influence of the westerly circulation, leading to a small difference in precipitation between cold and warm periods in the Late Pleistocene period [26], therefore, the population was maintained stably, until 7–5 Kya. Ili River population was expanded during the late Holocene period (6–1.5 Kya) with a relatively wet conditions induced by stronger westerly circulation [27]. BSPs pointed out all the populations experienced a depression in 19^th^ century when population explosion as well as the development of agriculture and industry caused by the urbanization along the rivers. The ecological consequences of human activities were water shortage, soil salinization, enhanced ecological degradation and desertification.

The collection of samples turned out to be very challenging in Kashgar and Hotan Rivers because of both environmental and climatic changes (aridification and desertification) as well as human activities such as overfishing, reservoirs, groundwater wells and hydropower stations. Especially, jade mining in Hotan River [19], changed the watercourse and destroyed freshwater habitats.

### Drainage history of Tarim River

Compared with the modern drainage patterns, the paleodrainage was in better agreement with the phylogeographic structure which aims to decode the history of species evolution, and to reconstruct the sequence of evolutionary events [17, 28, 29]. The current Tarim River has a five-source-one-mainstream pattern, i.e., the North Tarim River. However, it is a controversial view about the existence of the South Tarim River among scientists, some consider that the South Tarim River existed in history, but some others disagree [30]. We used a phylogeographic approach to assess the evolution and distribution patterns of freshwater fish *D. maculatus* generated from mtDNA data, which reflect the palaeogeographical complexity of a region, especially, the development of rivers and their isolation and interconnection processes [29]. Based on our analysis, populations from five tributaries of Tarim River were classified into two clades, the North clade containing three subclades (Weigan, Aksu, Kashgar), and the South one containing two subclades (Yarkand and Hotan), which were geographically divided by the Taklimakan Desert. Similarly, the nucDNA RAG-2 showed that the gene flow existed within the north or south clade only. It seemed that the disintegrated modern Tarim River system was inconsistent with the phylogeologic history of *D. maculatus*. Therefore, our observation supported the hypothesis that the South Tarim River has indeed existed in history. In the further analysis, more nucDNA markers are required to verify the hypothesis accurately.

Ayelhan et al. [31] proposed that *D. maculatus* originated from West Kunlun area. Amongst the five subclades of Tarim River, the highest values of *F*_ST_ and nucleotide diversity in Yarkand population suggested it was a possible center of the origin of the species.

## Methods

### Sampling and laboratory procedures

*D. maculatus* is a species under protection, hence difficult to sample [16, 17, 32]. The entire animal experiment was conducted according to the principles expressed in the “Guide for the Care and Use of Laboratory Animals” by National Research Council of the National Academies of Science. Samples were collected using gill nets or cast nets in June and July 2010, May to June in 2013, as well as July and August in 2014. All the specimens were preserved in 95 % ethanol for further laboratory analyses. The definition of samples was on the basis of the classical taxonomic description by Chen & Cao [17] and Wu & Wu [16]. A total of 261 individuals of *D. maculatus* were used for the phylogenetic and population genetic analyses (Additional file 2: Table S1). The samples were collected from 16 populations across three different river systems in northwestern China (Fig. 1), including 47 individuals from Ili River Basin, 34 individuals from Indus River Basin, and 180 individuals from Tarim River Basin (Table 1). According to the comparative morphology [16, 17] and molecular phylogenetics [33, 34], *Gymnodiptychus dybowskii* (GenBank accession number KJ081377 for 16S rRNA and KJ081423 for Cyt *b*) [22], *Aspiorhynchus laticeps* (KF564793) [35], and *Barbus barbus* (AB238965) [36] were used as outgroups in the phylogenetic analyses based on their locations in the Tarim River (Xinjiang), Tarim River (Xinjiang) and Danube River (Austria) respectively. Voucher specimens were deposited in the key Laboratory of Adaptation and Evolution of Plateau Biota, Northwest Plateau Institute of Biology, the Chinese Academy of Sciences in Xining (Additional file 2: Table S1).

Total genomic DNA was extracted with proteinase K followed by the standard 3-step phenol-chloroform method [37]. Complete sequence of Cyt *b* gene, partial sequences of the 16S rRNA, D-loop and RAG-2 genes were obtained for all the sampled individuals. When direct sequencing of RAG-2 failed due to heterozygosity, amplicons of RAG-2 were cloned using a TA-cloning system (TakaRa Biotechnology, Dalian, China) following the manufacturer’s instructions [38]. The master mixture of polymerase chain reaction (PCR) contained approximately 100 ng of template DNA, 1 μL (10 pmol) of each primer, 5 μL of 10× reaction buffer, 2 μL of dNTPs (2.5 mM of each) and 2.0 U of Taq DNA polymerase, in a total volume of 50 μL. Reactions were carried out on a Veriti Thermal Cycler (Applied Biosystems, Carlsbad, CA, USA) and always included a negative control. Individual thermal cycling parameters for each primer set are provided in Table 5, and all protocols began with 3 min at 95 °C and ended with 10 min at 72 °C. The amplified DNA was fractionated by electrophoresis through 0.8 % low-melting agarose gels, recovered from the gels, and purified with Gel Extraction Mini kit (Watson Biotechnologies, Shanghai, China). The purified DNA Sequencing was sequenced with the Perkin-Elmer BigDye DNA Sequencing Kit according to the manufacturer’s protocol, using the same primers employed in the PCR.

**Table 5:** Primers, corresponding references and PCR cycles used to Cyt *b*, 16S rRNA, D-loop and RAG-2

Locus	Primers	Primer reference	Thermal cycling protocol
Cyt *b*	L14724	[55]	[30 s at 94 °C, 30 s at 52 °C, 70 s at 72 °C] × 35
	H15915		
16S rRNA	16Sp1F	[56]	[30 s at 94 °C, 30 s at 48 °C, 90 s at 72 °C] × 33
	16Sp1R		
D-loop	GEDL200	[57]	[30 s at 94 °C, 30 s at 52 °C, 60 s at 72 °C] × 35
	GEDH860		
RAG-2	RAG2-f2	[58]	[60 s at 94 °C, 60 s at 60 °C, 105 s at 72 °C] × 35
	RAG2-R6		

### DNA sequence alignment

DNA sequences were edited using DNASTAR 5.0 (DNASTAR Inc.), and were aligned using CLUSTALX 2.0 as implemented in MEGA 5.05 with default parameters [39]. Identical haplotypes were collapsed using DNASP 5.1 [40].

### Phylogenetic analyses

Phylogenies of the mtDNA data were constructed using maximum likelihood (ML) and Bayesian inference (BI) as implemented in PHYML 3.0 [41] and MRBAYES 3.2.1 software [42]. The most appropriate nucleotide substitution models for the three segments were selected using Akaike Information Criterion as implemented in JMODELTEST 2.1.4 software [43]. Using a starting tree obtained by neighbour-joining. Clade robustness was assessed by bootstrap analysis using 1000 replicates. To assess the statistical significance of nodes, a bootstrap analysis with 100 replicates was used for the ML analyses, remaining settings set to default. The posterior distributions from BI were obtained by a Markov Chain Monte Carlo (MCMC) analysis with one cold chain and three heated chains. Samples of the trees and parameters were drawn every 100 steps from a total of 1 million MCMC generations. Three additional runs were conducted beginning with random trees. The 50 % majority rule consensus of the post-burn (using a burn-in of 25 %) for all the generations was computed for all the four runs.

The NETWORK 4.6 [44] was used to build a median-joining network for both mtDNA and nucDNA data.

### Molecular diversity and genetic structure

We used ARLEQUIN 3.5 software [45] for AMOVA and pairwise *F*_ST_ values. Both AMOVA and *F*_ST_ used Tamura & Nei genetic distance [46] with gamma correction for heterogeneity of mutation rates.

### Population demography

The time of divergence was estimated using a strict-clock Bayesian approach in BEASTv1.8.0 [47], with the GTR + G substitution model suggested by JMODELTEST 2.1.4 software [43], with Yule prior approach, and a random starting tree. In the absence of a fossil record of schizothoracines, we used the divergence-time estimates from He et al. [34] for an internal time calibration on branching point: *D. maculatus* vs. *G. dybowskii* (7.77 ± 0.51 Mya). Another calibration point was based on the European fossil records of the genus *Barbus*, species *B. barbus* from the Pliocene period (15–11 million years ago, Ma) [36, 48, 49]. The analysis included three independent MCMC runs sampled every 1000 generations for 30 million generations with 20 % of samples discarded as burn-in. The effective sample size for parameter estimates and convergence was checked using TRACER1.5 [50]. The corresponding tree files were merged with LOGCOMBINER v1.8.0, and trees were summarized using TREEANNOTATOR v1.8.0, two software programs distributed within the BEAST package [47]. Trees were visualized in FIGTREE 1.4 [51].

We used three different approaches to assess demographic history. These included two neutrality statistics, Tajima’s D and Fu’s *F*s [52, 53], calculated with 5000 permutations in ARLEQUIN 3.5 [54]. The negative values for these indices are consistent with population expansion. To evaluate the timing of any expansion, we used BSP obtained with BEASTv1.8.0 [47] derived with three independent runs (30 million simulations; first 10 % as burn-in). Both log files and tree files were analyzed using TRACER1.5 [50]; effective sample size for all parameters was more than 200. The results were consistent across runs. We then ran these analyses under the GTR + G model, with a strict molecular clock. The substitution rate for combined three mtDNA genes was estimated from the previously described analysis of divergence times to be 0.82 % per Mya with a 95 % highest posterior density (HPD) of (0.69, 0.91). We could not run BSPs for Kashgar and Hotan River population due to the small number of samples available (<10).

## Additional files

Additional file 1: Figure S1. Tarim River system in the late Pleistocene [30]. 1-Mountainous Region, 2-Tarim River Basin, 3-Ancient Lakes. (DOCX 503 kb) Additional file 2: Table S1. Detailed information for specimens of *Diptychus maculatus* included in this study. (DOCX 46 kb) Additional file 3: Table S2. The *F*_ST_ values among the populations of *Diptychus maculatus* based on the combined mtDNA data. (DOCX 21 kb) Additional file 4: Table S3. Mean divergence values between clades for *Diptychus maculatus*. (DOCX 16 kb) Additional file 5: Figure S2. Estimates of *Diptychus maculatus* divergence times obtained with BEASTv1.8.0. (DOCX 86 kb)

## Abbreviations

16S rRNA: 16S ribosomal RNA
AMOVA: Analyses of molecular variance
BSP: Bayesian skyline plots
Cyt *b*: Cytochrome *b*
*F*_ST_: Pairwise comparisons of genetic differentiation
HPD: Highest posterior density
MCMC: Markov Chain Monte Carlo
ML: Maximum likelihood
mtDNA: Mitochondrial DNA
NTS: North Tien Shan
PCR: Polymerase chain reaction
QTP: The Qinghai-Tibetan Plateau
RAG-2: Recombination activating gene 2
STS: South Tien Shan
TMRCA: Time to the most recent common ancestor

## References

[1] Li JJ, Fang XM (1999). Uplift of the Tibetan Plateau and environmental changes. Chin Sci Bull.

[2] Zhang DF, Fengquan L, Jianmin B (2000). Eco-environmental effects of the Qinghai-Tibet Plateau uplift during the Quaternary in China. Environ Geol.

[3] Shi YF, Zheng BX, Li SJ, Ye BS (1995). Studies on altitude and climatic environment in the middle and east parts of Tibetan Plateau during Quaternary Maximum Glaciation. J Glaciol Geocryol.

[4] Zhou SZ, Wang XL, Wang J, Xu LB (2006). A preliminary study on timing of the oldest Pleistocene glaciation in Qinghai-Tibetan Plateau. Quat Int.

[5] Guo Z, Zhang Z, Wu C, Fang S, Zhang R (2006). The Mesozoic and Cenozoic exhumation history of Tianshan and comparative studies to the Junggar and Altai Mountains. Acta Geol Sin.

[6] Li XM, Jiang FQ, Li LH, Wang GQ (2011). Spatial and temporal variability of precipitation concentration index, concentration degree and concentration period in Xinjiang, China. Int J Climatol.

[7] Fang XM, Lu LQ, Yang SL, Li JJ, An ZS, Jiang P, Chen XL (2002). Loess in Kunlun Mountains and its implications on desert development and Tibetan Plateau uplift in west China. Sci China Ser D.

[8] Zhang QS, Li BY (1989). A preliminary study on the uplifting and environment evolution of the Karakoram and West Kunlun Mountains area since late Cenozoic era. J Nat Resour.

[9] Platnick NI, Nelson G (1984). Composite areas in vicariance biogeography. Syst Zool.

[10] He DK, Chen YF (2007). Molecular phylogeny and biogeography of the highly specialized grade schizothoracine fishes (Teleostei : Cyprinidae) inferred from cytochrome b sequences. Chin Sci Bull.

[11] Berendzen PB, Simons AM, Wood RM (2003). Phylogeography of the northern hogsucker, *Hypentelium nigricans* (Teleostei: Cypriniformes): genetic evidence for the existence of the ancient Teays River. J Biogeogr.

[12] Hurwood DA, Hughes JM (1998). Phylogeography of the freshwater fish, *Mogurnda adspersa*, in streams of northeastern Queensland, Australia: evidence for altered drainage patterns. Mol Ecol.

[13] Montoya-Burgos J (2003). Historical biogeography of the catfish genus *Hypostomus* (Siluriformes: Loricariidae), with implications on the diversification of Neotropical ichthyofauna. Mol Ecol.

[14] Cao WX, Chen YY, Wu YF, Zhu SQ (1981). Origin and evolution of schizothoracine fishes in relation to the upheaval of the Xizang Plateau. Studies on the period, amplitude and type of the uplift of the Qinghai-Xizang Plateau.

[15] Zhang DR, Chen MY, Murphy RW, Che J, Pang JF, Hu JS, Luo J, Wu SJ, Ye H, Zhang YP (2010). Genealogy and palaeodrainage basins in Yunnan Province: phylogeography of the Yunnan spiny frog, Nanorana yunnanensis (Dicroglossidae). Mol Ecol.

[16] Wu YF, Wu CZ (1992). The fishes of the Qinghai-Xizang plateau.

[17] Chen YF, Cao WX (2000). Schizothoracinae. Fauna Sinica, Osteichthyes, Cypriniformes III.

[18] Guo Y, Meng W, Liu J, Zhang RM, Cai LG (2009). Comparison of morphological characters of *Diptychus maculates* in different rivers of Xinjiang. Chinese J Fish.

[19] Meng W, Yang TY, Hai S, Ma YW, Cai LG, Ma XF, Gao TX, Guo Y (2015). Extensive genetic divergence among *Diptychus maculatus* populations in northwest China. Chin J Oceanol Limnol.

[20] Yang TY, Meng W, Ma YW, Cai LG, Guo Y (2014). Genetic structure analysis of *Diptychus maculatus* between two water systems in Xinjiang based on mitochondrial COI and Cyt *b* gene sequences. Freshw Fish.

[21] Fijarczyk A, Nadachowska K, Hofman S, Litvinchuk SN, Babik W, Stuglik M, Gollmann G, Choleva L, Cogalniceanu D, Vukov T (2011). Nuclear and mitochondrial phylogeography of the European fire-bellied toads *Bombina bombina* and *Bombina variegata* supports their independent histories. Mol Ecol.

[22] Li GG, Peng ZG, Zhang RY, Tang YT, Tong C, Feng CC, Zhang CF, Zhao K (2016). Mito-nuclear phylogeography of the cyprinid fish *Gymnodiptychus dybowskii* in the arid Tien Shan region of Central Asia. Biol J Linn Soc.

[23] Wielstra B, Babik W, Arntzen JW (2015). The crested newt *Triturus cristatus* recolonized temperate Eurasia from an extra-Mediterranean glacial refugium. Biol J Linn Soc.

[24] Meng HH, Zhang ML (2013). Diversification of plant species in arid Northwest China: species-level phylogeographical history of *Lagochilus* Bunge ex Bentham (Lamiaceae). Mol Phylogenet Evol.

[25] Yang XP, Scuderi LA (2010). Hydrological and climatic changes in deserts of China since the late Pleistocene. Quat Res.

[26] ChongYi E, Lai ZP, Sun YJ, Hou GL, Yu LP, Wu CY (2012). A luminescence dating study of loess deposits from the Yili River basin in western China. Quat Geochronol.

[27] Huang XZ, Chen FH, Fan YX, Yang ML (2009). Dry late-glacial and early Holocene climate in arid central Asia indicated by lithological and palynological evidence from Bosten Lake, China. Quat Int.

[28] Deagle BE, Jones FC, Absher DM, Kingsley DM, Reimchen TE (2013). Phylogeography and adaptation genetics of stickleback from the Haida Gwaii archipelago revealed using genome-wide single nucleotide polymorphism genotyping. Mol Ecol.

[29] Bernatchez L, Wilson CC (1998). Comparative phylogeography of nearctic and palearctic fishes. Mol Ecol.

[30] Liu Y, Tian F, Hu H, Sivapalan M (2014). Socio-hydrologic perspectives of the co-evolution of humans and water in the Tarim River basin, Western China: the Taiji-Tire model. Hydrol Earth Syst Sc.

[31] Ayelhan H, Guo Y, Meng W, Yang T, Ma Y (2014). Phylogeny and divergence time estimation of Schizothoracinae fishes in Xinjiang. Hereditas.

[32] Ma YW, Guo Y, Zhang RM, Tu EX, Xie CG, Liu J, Li L (2009). Fauna composition and distribution of aboriginal fish in the Tarim River of Xinjiang Uygur Autonomous Region. J Fish China.

[33] Qi DL, Chao Y, Guo SC, Zhao LY, Li TP, Wei FL, Zhao XQ (2012). Convergent, parallel and correlated evolution of trophic morphologies in the subfamily Schizothoracinae from the Qinghai-Tibetan plateau. PLoS One.

[34] He DK, Chen YF, Chen YY, Chen ZM (2004). Molecular phylogeny of the specialized schizothoracine fishes (Teleostei: Cyprinidae), with their implications for the uplift of the Qinghai-Tibetan Plateau. Chin Sci Bull.

[35] Jiang M, Yang CG, Wen H (2014). The complete mitochondrial genome of *Aspiorhynchus laticeps* and its phylogenetic analysis. Meta Gene.

[36] Saitoh K, Sado T, Mayden RL, Hanzawa N, Nakamura K, Nishida M, Miya M (2006). Mitogenomic evolution and interrelationships of the cypriniformes (Actinopterygii: Ostariophysi): The first evidence toward resolution of higher-level relationships of the world’s largest freshwater fish clade based on 59 whole mitogenome sequences. J Mol Evol.

[37] Sambrook J, Fritsch EF, Maniatis T (1989). Molecular cloning.

[38] Cheang CC, Chu KH, Ang PO (2010). Phylogeography of the marine macroalga *Sargassum hemiphyllum* (Phaeophyceae, Heterokontophyta) in northwestern Pacific. Mol Ecol.

[39] Tamura K, Peterson D, Peterson N, Stecher G, Nei M, Kumar S (2011). MEGA5: molecular evolutionary genetics analysis using maximum likelihood, evolutionary distance, and maximum parsimony methods. Mol Biol Evol.

[40] Librado P, Rozas J (2009). DnaSP v5: a software for comprehensive analysis of DNA polymorphism data. Bioinformatics.

[41] Guindon S, Dufayard JF, Lefort V, Anisimova M, Hordijk W, Gascuel O (2010). New algorithms and methods to estimate maximum-likelihood phylogenies: assessing the performance of PhyML 3.0. Syst Biol.

[42] Ronquist F, Teslenko M, van der Mark P, Ayres DL, Darling A, Hohna S, Larget B, Liu L, Suchard MA, Huelsenbeck JP (2012). MrBayes 3.2: efficient Bayesian phylogenetic inference and model choice across a large model space. Syst Biol.

[43] Darriba D, Taboada GL, Doallo R, Posada D (2012). jModelTest 2: more models, new heuristics and parallel computing. Nat Methods.

[44] Bandelt HJ, Forster P, Rohl A. Median-joining networks for inferring intraspecific phylogenies. Mol Biol Evol. 1999;16:37–48. 10.1093/oxfordjournals.molbev.a026036 10331250

[45] Excoffier L, Smouse PE, Quattro JM (1992). Analysis of molecular variance inferred from metric distances among DNA haplotypes-application to human mitochondrial-DNA restriction data. Genetics.

[46] Tamura K, Nei M (1993). Estimation of the number of nucleotide substitutions in the control region of mitochondrial-DNA in humans and chimpanzees. Mol Biol Evol.

[47] Drummond AJ, Suchard MA, Xie D, Rambaut A (2012). Bayesian phylogenetics with BEAUti and the BEAST 1.7. Mol Biol Evol.

[48] Zardoya R, Doadrio I (1999). Molecular evidence on the evolutionary and biogeographical patterns of European cyprinids. J Mol Evol.

[49] Zhao K, Duan ZY, Peng ZG, Guo SC, Li JB, He SP, Zhao XQ (2009). The youngest split in sympatric schizothoracine fish (Cyprinidae) is shaped by ecological adaptations in a Tibetan Plateau glacier lake. Mol Ecol.

[50] Rambaut A, Drummond A. Tracer v1. 4th edition. 2007. http://beast.bio.ed.ac.uk/Tracer.

[51] Rambaut A, Drummond A. FigTree version 1.4. 2012. Available at: http://tree.bio.ed.ac.uk/software/figtree.

[52] Fu YX (1997). Statistical tests of neutrality of mutations against population growth, hitchhiking and background selection. Genetics.

[53] Tajima F (1989). Statistical-method for testing the neutral mutation hypothesis by DNA polymorphism. Genetics.

[54] Excoffier L, Lischer HEL (2010). Arlequin suite ver 3.5: a new series of programs to perform population genetics analyses under Linux and Windows. Mol Ecol Resour.

[55] Xiao WH, Zhang YP, Liu HZ (2001). Molecular systematics of Xenocyprinae (Teleostei: Cyprinidae): taxonomy, biogeography, and coevolution of a special group restricted in East Asia. Mol Phylogenet Evol.

[56] Li JB, Wang XZ, Kong XH, Zhao K, He SP, Mayden RL (2008). Variation patterns of the mitochondrial 16S rRNA gene with secondary structure constraints and their application to phylogeny of cyprinine fishes (Teleostei: Cypriniformes). Mol Phylogenet Evol.

[57] Zhao K, Duan ZY, Peng ZG, Gan XN, Zhang RY, He SP, Zhao XQ (2011). Phylogeography of the endemic *Gymnocypris chilianensis* (Cyprinidae): sequential westward colonization followed by allopatric evolution in response to cyclical Pleistocene glaciations on the Tibetan Plateau. Mol Phylogenet Evol.

[58] Lovejoy NR, Collette BB. Phylogenetic relationships of new world needlefishes (Teleostei: Belonidae) and the biogeography of transitions between marine and freshwater habitats. Copeia. 2001;324–338.

