# Supplementary figures and images for: Phylogeography of Diptychus maculatus (Cyprinidae) endemic to the northern margin of the QTP and Tien Shan region
Source: BMC Evol Biol. 2016 Sep 9;16(1):186. doi: 10.1186/s12862-016-0756-3 (PMC5017051; doi:10.1186/s12862-016-0756-3)

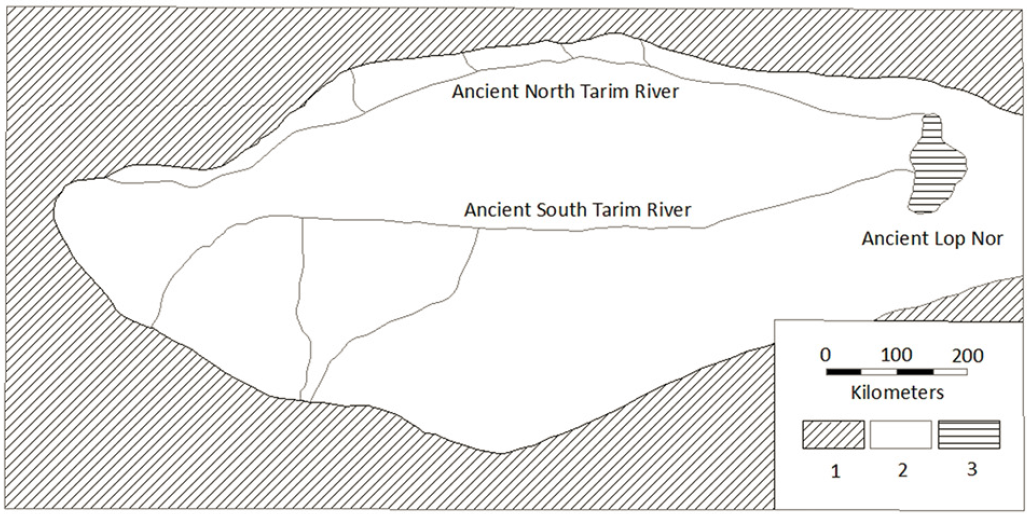


**Figure S1**

Supplement: Additional file 1: Figure S1. — Tarim River system in the late Pleistocene [30]. 1-Mountainous Region, 2-Tarim River Basin, 3-Ancient Lakes. (DOCX 503 kb) [file 12862_2016_756_MOESM1_ESM.docx]

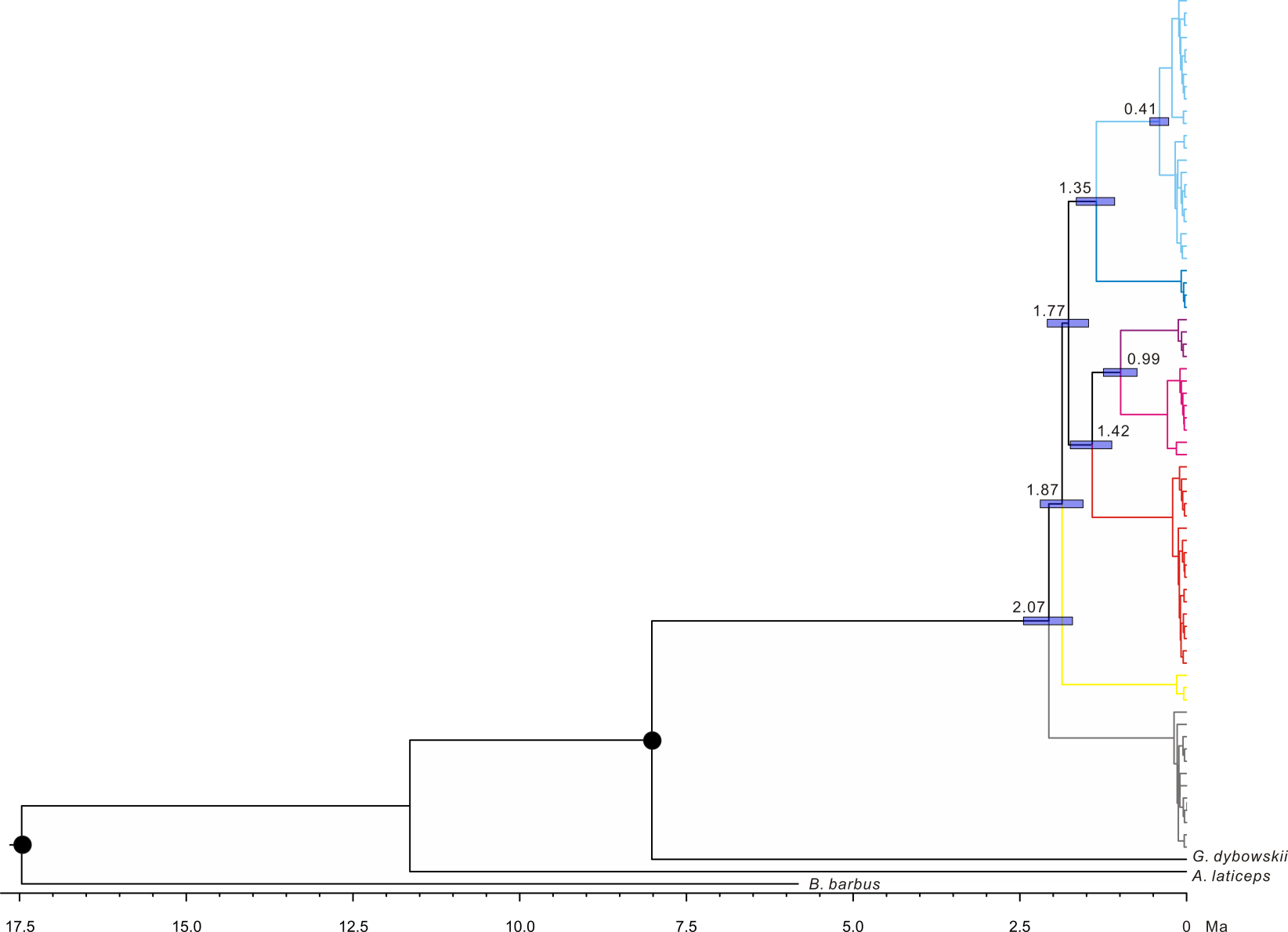


**Figure S2**

Supplement: Additional file 5: Figure S2. — Estimates of Diptychus maculatus divergence times obtained with BEASTv1.8.0. (DOCX 86 kb) [file 12862_2016_756_MOESM5_ESM.docx]
