# Supplementary material for: Phylogeography of Diptychus maculatus (Cyprinidae) endemic to the northern margin of the QTP and Tien Shan region
Source: BMC Evol Biol. 2016 Sep 9;16(1):186. doi: 10.1186/s12862-016-0756-3 (PMC5017051; doi:10.1186/s12862-016-0756-3)
Supplement: Additional file 2: Table S1. — Detailed information for specimens of Diptychus maculatus included in this study. (DOCX 46 kb) [file 12862_2016_756_MOESM2_ESM.docx]

**Table S1** Detailed information for specimens included in this study.

| **Species name** | **Haplotype**  **(mtDNA)** | **Haplotype**  **(nuDNA)** | **Sample number** | **GenBank Accession No.** | | | | **Clades/Subclades** | **Locality codes** |
| --- | --- | --- | --- | --- | --- | --- | --- | --- | --- |
|  |  |  |  | **Cyt *b*** | **16SrRNA** | **D-loop** | **RAG-2** |  |  |
| Ingroup |  |  |  |  |  |  |  |  |  |
| *D.maculatus* | IL1 | R6 | 1305134 | KX022699 | KX022736 | KX022767 | KX022728 | Ili River | 2 |
| *D.maculatus* | IL1 | R6 | 1305135 | KX022699 | KX022736 | KX022767 | KX022728 | Ili River | 2 |
| *D.maculatus* | IL1 | R6 | 1305136 | KX022699 | KX022736 | KX022767 | KX022728 | Ili River | 2 |
| *D.maculatus* | IL1 | R6 | 1305137 | KX022699 | KX022736 | KX022767 | KX022728 | Ili River | 2 |
| *D.maculatus* | IL1 | R6 | 1305148 | KX022699 | KX022736 | KX022767 | KX022728 | Ili River | 2 |
| *D.maculatus* | IL1 | R6 | 1305149 | KX022699 | KX022736 | KX022767 | KX022728 | Ili River | 2 |
| *D.maculatus* | IL1 | R6 | 1305150 | KX022699 | KX022736 | KX022767 | KX022728 | Ili River | 2 |
| *D.maculatus* | IL1 | R6 | 1305151 | KX022699 | KX022736 | KX022767 | KX022728 | Ili River | 2 |
| *D.maculatus* | IL1 | R6 | 1305156 | KX022699 | KX022736 | KX022767 | KX022728 | Ili River | 2 |
| *D.maculatus* | IL1 | R6 | 1305157 | KX022699 | KX022736 | KX022767 | KX022728 | Ili River | 2 |
| *D.maculatus* | IL1 | R6 | 1305158 | KX022699 | KX022736 | KX022767 | KX022728 | Ili River | 2 |
| *D.maculatus* | IL1 | R6 | 1305159 | KX022699 | KX022736 | KX022767 | KX022728 | Ili River | 2 |
| *D.maculatus* | IL1 | R6 | 1007258 | KX022699 | KX022736 | KX022767 | KX022728 | Ili River | 2 |
| *D.maculatus* | IL1 | R6 | 1007260 | KX022699 | KX022736 | KX022767 | KX022728 | Ili River | 2 |
| *D.maculatus* | IL2 | R6 | 1305051 | KX022699 | KX022737 | KX022768 | KX022728 | Ili River | 1 |
| *D.maculatus* | IL2 | R6 | 1305056 | KX022699 | KX022737 | KX022768 | KX022728 | Ili River | 1 |
| *D.maculatus* | IL2 | R6 | 1305057 | KX022699 | KX022737 | KX022768 | KX022728 | Ili River | 1 |
| *D.maculatus* | IL2 | R6 | 1305060 | KX022699 | KX022737 | KX022768 | KX022728 | Ili River | 1 |
| *D.maculatus* | IL2 | R6 | 1305061 | KX022699 | KX022737 | KX022768 | KX022728 | Ili River | 1 |
| *D.maculatus* | IL2 | R6 | 1305063 | KX022699 | KX022737 | KX022768 | KX022728 | Ili River | 1 |
| *D.maculatus* | IL2 | R6 | 1305066 | KX022699 | KX022737 | KX022768 | KX022728 | Ili River | 1 |
| *D.maculatus* | IL3 | R6 | 1305052 | KX022699 | KX022738 | KX022769 | KX022728 | Ili River | 1 |
| *D.maculatus* | IL3 | R6 | 1305054 | KX022699 | KX022738 | KX022769 | KX022728 | Ili River | 1 |
| *D.maculatus* | IL3 | R6 | 1305062 | KX022699 | KX022738 | KX022769 | KX022728 | Ili River | 1 |
| *D.maculatus* | IL3 | R6 | 1305064 | KX022699 | KX022738 | KX022769 | KX022728 | Ili River | 1 |
| *D.maculatus* | IL3 | R6 | 1305065 | KX022699 | KX022738 | KX022769 | KX022728 | Ili River | 1 |
| *D.maculatus* | IL4 | R6 | 1305055 | KX022699 | KX022738 | KX022767 | KX022728 | Ili River | 1 |
| *D.maculatus* | IL4 | R6 | 1305069 | KX022699 | KX022738 | KX022767 | KX022728 | Ili River | 1 |
| *D.maculatus* | IL5 | R6 | 1305058 | KX022699 | KX022738 | KX022770 | KX022728 | Ili River | 1 |
| *D.maculatus* | IL6 | R6 | 1305068 | KX022699 | KX022738 | KX022771 | KX022728 | Ili River | 1 |
| *D.maculatus* | IL7 | R6 | 1305133 | KX022700 | KX022738 | KX022767 | KX022728 | Ili River | 2 |
| *D.maculatus* | IL7 | R6 | 1305138 | KX022700 | KX022738 | KX022767 | KX022728 | Ili River | 2 |
| *D.maculatus* | IL7 | R6 | 1305140 | KX022700 | KX022738 | KX022767 | KX022728 | Ili River | 2 |
| *D.maculatus* | IL7 | R6 | 1305147 | KX022700 | KX022738 | KX022767 | KX022728 | Ili River | 2 |
| *D.maculatus* | IL7 | R6 | 1305153 | KX022700 | KX022738 | KX022767 | KX022728 | Ili River | 2 |
| *D.maculatus* | IL7 | R6 | 1305154 | KX022700 | KX022738 | KX022767 | KX022728 | Ili River | 2 |
| *D.maculatus* | IL7 | R6 | 1305155 | KX022700 | KX022738 | KX022767 | KX022728 | Ili River | 2 |
| *D.maculatus* | IL7 | R6 | 1305160 | KX022700 | KX022738 | KX022767 | KX022728 | Ili River | 2 |
| *D.maculatus* | IL7 | R6 | 1305161 | KX022700 | KX022738 | KX022767 | KX022728 | Ili River | 2 |
| *D.maculatus* | IL8 | R6 | 1305139 | KX022699 | KX022736 | KX022772 | KX022728 | Ili River | 2 |
| *D.maculatus* | IL9 | R6 | 1305141 | KX022699 | KX022738 | KX022773 | KX022728 | Ili River | 3 |
| *D.maculatus* | IL9 | R6 | 1305144 | KX022699 | KX022738 | KX022773 | KX022728 | Ili River | 3 |
| *D.maculatus* | IL9 | R6 | 1305145 | KX022699 | KX022738 | KX022773 | KX022728 | Ili River | 3 |
| *D.maculatus* | IL9 | R6 | 1305146 | KX022699 | KX022738 | KX022773 | KX022728 | Ili River | 3 |
| *D.maculatus* | IL10 | R6 | 1305142 | KX022699 | KX022739 | KX022773 | KX022728 | Ili River | 3 |
| *D.maculatus* | IL11 | R6 | 1305143 | KX022699 | KX022738 | KX022774 | KX022728 | Ili River | 3 |
| *D.maculatus* | IL12 | R6 | 1305152 | KX022701 | KX022736 | KX022767 | KX022728 | Ili River | 2 |
| *D.maculatus* | W1 | R1 | 1305164 | KX022702 | KX022740 | KX022775 | KX022729 | North Tarim /Weigan River | 6 |
| *D.maculatus* | W1 | R1 | 1305165 | KX022702 | KX022740 | KX022775 | KX022729 | North Tarim /Weigan River | 6 |
| *D.maculatus* | W1 | R1 | 1305167 | KX022702 | KX022740 | KX022775 | KX022729 | North Tarim /Weigan River | 6 |
| *D.maculatus* | W1 | R1 | 1305175 | KX022702 | KX022740 | KX022775 | KX022729 | North Tarim /Weigan River | 6 |
| *D.maculatus* | W1 | R1 | 1305178 | KX022702 | KX022740 | KX022775 | KX022729 | North Tarim /Weigan River | 6 |
| *D.maculatus* | W1 | R1 | 1305180 | KX022702 | KX022740 | KX022775 | KX022729 | North Tarim /Weigan River | 6 |
| *D.maculatus* | W1 | R1 | 1305188 | KX022702 | KX022740 | KX022775 | KX022729 | North Tarim /Weigan River | 6 |
| *D.maculatus* | W1 | R1 | 1305189 | KX022702 | KX022740 | KX022775 | KX022729 | North Tarim /Weigan River | 6 |
| *D.maculatus* | W1 | R1 | 1305191 | KX022702 | KX022740 | KX022775 | KX022729 | North Tarim /Weigan River | 6 |
| *D.maculatus* | W1 | R1 | 1305193 | KX022702 | KX022740 | KX022775 | KX022729 | North Tarim /Weigan River | 6 |
| *D.maculatus* | W1 | R1 | 1305197 | KX022702 | KX022740 | KX022775 | KX022729 | North Tarim /Weigan River | 6 |
| *D.maculatus* | W1 | R1 | 1305199 | KX022702 | KX022740 | KX022775 | KX022729 | North Tarim /Weigan River | 6 |
| *D.maculatus* | W1 | R1 | 1305200 | KX022702 | KX022740 | KX022775 | KX022729 | North Tarim /Weigan River | 6 |
| *D.maculatus* | W1 | R1 | 1305201 | KX022702 | KX022740 | KX022775 | KX022729 | North Tarim /Weigan River | 6 |
| *D.maculatus* | W1 | R1 | 1305203 | KX022702 | KX022740 | KX022775 | KX022729 | North Tarim /Weigan River | 6 |
| *D.maculatus* | W1 | R1 | 1305206 | KX022702 | KX022740 | KX022775 | KX022729 | North Tarim /Weigan River | 6 |
| *D.maculatus* | W1 | R1 | 1305207 | KX022702 | KX022740 | KX022775 | KX022729 | North Tarim /Weigan River | 6 |
| *D.maculatus* | W1 | R1 | 1305211 | KX022702 | KX022740 | KX022775 | KX022729 | North Tarim /Weigan River | 6 |
| *D.maculatus* | W1 | R1 | 1305214 | KX022702 | KX022740 | KX022775 | KX022729 | North Tarim /Weigan River | 7 |
| *D.maculatus* | W1 | R1 | 1305218 | KX022702 | KX022740 | KX022775 | KX022729 | North Tarim /Weigan River | 7 |
| *D.maculatus* | W1 | R1 | 1305220 | KX022702 | KX022740 | KX022775 | KX022729 | North Tarim /Weigan River | 7 |
| *D.maculatus* | W1 | R1 | 1305221 | KX022702 | KX022740 | KX022775 | KX022729 | North Tarim /Weigan River | 7 |
| *D.maculatus* | W1 | R1 | 1305222 | KX022702 | KX022740 | KX022775 | KX022729 | North Tarim /Weigan River | 7 |
| *D.maculatus* | W1 | R1 | 1305225 | KX022702 | KX022740 | KX022775 | KX022729 | North Tarim /Weigan River | 7 |
| *D.maculatus* | W1 | R1 | 1305226 | KX022702 | KX022740 | KX022775 | KX022729 | North Tarim /Weigan River | 7 |
| *D.maculatus* | W1 | R1 | 1305239 | KX022702 | KX022740 | KX022775 | KX022729 | North Tarim /Weigan River | 7 |
| *D.maculatus* | W1 | R1 | 1305251 | KX022702 | KX022740 | KX022775 | KX022729 | North Tarim /Weigan River | 7 |
| *D.maculatus* | W1 | R1 | 1305255 | KX022702 | KX022740 | KX022775 | KX022729 | North Tarim /Weigan River | 7 |
| *D.maculatus* | W2 | R1 | 1305168 | KX022702 | KX022740 | KX022776 | KX022729 | North Tarim /Weigan River | 6 |
| *D.maculatus* | W2 | R1 | 1305169 | KX022702 | KX022740 | KX022776 | KX022729 | North Tarim /Weigan River | 6 |
| *D.maculatus* | W2 | R1 | 1305172 | KX022702 | KX022740 | KX022776 | KX022729 | North Tarim /Weigan River | 6 |
| *D.maculatus* | W2 | R1 | 1305181 | KX022702 | KX022740 | KX022776 | KX022729 | North Tarim /Weigan River | 6 |
| *D.maculatus* | W2 | R1 | 1305187 | KX022702 | KX022740 | KX022776 | KX022729 | North Tarim /Weigan River | 6 |
| *D.maculatus* | W2 | R1 | 1305202 | KX022702 | KX022740 | KX022776 | KX022729 | North Tarim /Weigan River | 6 |
| *D.maculatus* | W2 | R1 | 1305209 | KX022702 | KX022740 | KX022776 | KX022729 | North Tarim /Weigan River | 6 |
| *D.maculatus* | W2 | R1 | 1305213 | KX022702 | KX022740 | KX022776 | KX022729 | North Tarim /Weigan River | 7 |
| *D.maculatus* | W2 | R1 | 1305223 | KX022702 | KX022740 | KX022776 | KX022729 | North Tarim /Weigan River | 7 |
| *D.maculatus* | W2 | R1 | 1305227 | KX022702 | KX022740 | KX022776 | KX022729 | North Tarim /Weigan River | 7 |
| *D.maculatus* | W2 | R1 | 1305247 | KX022702 | KX022740 | KX022776 | KX022729 | North Tarim /Weigan River | 7 |
| *D.maculatus* | W2 | R1 | 1305248 | KX022702 | KX022740 | KX022776 | KX022729 | North Tarim /Weigan River | 7 |
| *D.maculatus* | W2 | R1 | 1305249 | KX022702 | KX022740 | KX022776 | KX022729 | North Tarim /Weigan River | 7 |
| *D.maculatus* | W3 | R1 | 1305171 | KX022703 | KX022740 | KX022775 | KX022729 | North Tarim /Weigan River | 6 |
| *D.maculatus* | W3 | R1 | 1305173 | KX022703 | KX022740 | KX022775 | KX022729 | North Tarim /Weigan River | 6 |
| *D.maculatus* | W3 | R1 | 1305182 | KX022703 | KX022740 | KX022775 | KX022729 | North Tarim /Weigan River | 6 |
| *D.maculatus* | W3 | R1 | 1305217 | KX022703 | KX022740 | KX022775 | KX022729 | North Tarim /Weigan River | 7 |
| *D.maculatus* | W3 | R1 | 1305224 | KX022703 | KX022740 | KX022775 | KX022729 | North Tarim /Weigan River | 7 |
| *D.maculatus* | W4 | R1 | 1305174 | KX022704 | KX022740 | KX022777 | KX022729 | North Tarim /Weigan River | 6 |
| *D.maculatus* | W4 | R1 | 1305177 | KX022704 | KX022740 | KX022777 | KX022729 | North Tarim /Weigan River | 6 |
| *D.maculatus* | W4 | R1 | 1305179 | KX022704 | KX022740 | KX022777 | KX022729 | North Tarim /Weigan River | 6 |
| *D.maculatus* | W4 | R1 | 1305183 | KX022704 | KX022740 | KX022777 | KX022729 | North Tarim /Weigan River | 6 |
| *D.maculatus* | W4 | R1 | 1305186 | KX022704 | KX022740 | KX022777 | KX022729 | North Tarim /Weigan River | 6 |
| *D.maculatus* | W4 | R1 | 1305210 | KX022704 | KX022740 | KX022777 | KX022729 | North Tarim /Weigan River | 6 |
| *D.maculatus* | W4 | R1 | 1305212 | KX022704 | KX022740 | KX022777 | KX022729 | North Tarim /Weigan River | 7 |
| *D.maculatus* | W4 | R1 | 1305216 | KX022704 | KX022740 | KX022777 | KX022729 | North Tarim /Weigan River | 7 |
| *D.maculatus* | W4 | R1 | 1305230 | KX022704 | KX022740 | KX022777 | KX022729 | North Tarim /Weigan River | 7 |
| *D.maculatus* | W4 | R1 | 1305240 | KX022704 | KX022740 | KX022778 | KX022729 | North Tarim /Weigan River | 7 |
| *D.maculatus* | W4 | R1 | 1305250 | KX022704 | KX022740 | KX022777 | KX022729 | North Tarim /Weigan River | 7 |
| *D.maculatus* | W4 | R1 | 1305254 | KX022704 | KX022740 | KX022777 | KX022729 | North Tarim /Weigan River | 7 |
| *D.maculatus* | W4 | R1 | 1305256 | KX022704 | KX022740 | KX022777 | KX022729 | North Tarim /Weigan River | 7 |
| *D.maculatus* | W5 | R1 | 1305176 | KX022705 | KX022740 | KX022775 | KX022729 | North Tarim /Weigan River | 6 |
| *D.maculatus* | W5 | R1 | 1305234 | KX022705 | KX022740 | KX022775 | KX022729 | North Tarim /Weigan River | 7 |
| *D.maculatus* | W6 | R1 | 1305184 | KX022702 | KX022741 | KX022776 | KX022729 | North Tarim /Weigan River | 6 |
| *D.maculatus* | W7 | R1 | 1305185 | KX022706 | KX022740 | KX022775 | KX022729 | North Tarim /Weigan River | 6 |
| *D.maculatus* | W7 | R1 | 1305208 | KX022706 | KX022740 | KX022775 | KX022729 | North Tarim /Weigan River | 6 |
| *D.maculatus* | W8 | R1 | 1305190 | KX022702 | KX022742 | KX022775 | KX022729 | North Tarim /Weigan River | 6 |
| *D.maculatus* | W8 | R1 | 1305233 | KX022702 | KX022742 | KX022775 | KX022729 | North Tarim /Weigan River | 7 |
| *D.maculatus* | W9 | R1 | 1305192 | KX022705 | KX022740 | KX022778 | KX022729 | North Tarim /Weigan River | 6 |
| *D.maculatus* | W9 | R1 | 1305204 | KX022705 | KX022740 | KX022778 | KX022729 | North Tarim /Weigan River | 6 |
| *D.maculatus* | W9 | R1 | 1305215 | KX022705 | KX022740 | KX022778 | KX022729 | North Tarim /Weigan River | 7 |
| *D.maculatus* | W9 | R1 | 1305228 | KX022705 | KX022740 | KX022778 | KX022729 | North Tarim /Weigan River | 7 |
| *D.maculatus* | W9 | R1 | 1305232 | KX022705 | KX022740 | KX022778 | KX022729 | North Tarim /Weigan River | 7 |
| *D.maculatus* | W9 | R1 | 1305235 | KX022705 | KX022740 | KX022778 | KX022729 | North Tarim /Weigan River | 7 |
| *D.maculatus* | W9 | R1 | 1305237 | KX022705 | KX022740 | KX022778 | KX022729 | North Tarim /Weigan River | 7 |
| *D.maculatus* | W9 | R1 | 1305252 | KX022705 | KX022740 | KX022778 | KX022729 | North Tarim /Weigan River | 7 |
| *D.maculatus* | W9 | R1 | 1305253 | KX022705 | KX022740 | KX022778 | KX022729 | North Tarim /Weigan River | 7 |
| *D.maculatus* | W10 | R1 | 1305194 | KX022707 | KX022740 | KX022777 | KX022729 | North Tarim /Weigan River | 6 |
| *D.maculatus* | W10 | R1 | 1305195 | KX022707 | KX022740 | KX022777 | KX022729 | North Tarim /Weigan River | 6 |
| *D.maculatus* | W11 | R1 | 1305196 | KX022702 | KX022743 | KX022776 | KX022729 | North Tarim /Weigan River | 6 |
| *D.maculatus* | W12 | R1 | 1305198 | KX022708 | KX022740 | KX022778 | KX022729 | North Tarim /Weigan River | 6 |
| *D.maculatus* | W13 | R1 | 1305219 | KX022704 | KX022744 | KX022777 | KX022729 | North Tarim /Weigan River | 7 |
| *D.maculatus* | W13 | R1 | 1305244 | KX022704 | KX022744 | KX022777 | KX022729 | North Tarim /Weigan River | 7 |
| *D.maculatus* | W13 | R1 | 1305257 | KX022704 | KX022744 | KX022777 | KX022729 | North Tarim /Weigan River | 7 |
| *D.maculatus* | W14 | R1 | 1305229 | KX022705 | KX022740 | KX022779 | KX022729 | North Tarim /Weigan River | 7 |
| *D.maculatus* | W15 | R1 | 1305231 | KX022709 | KX022740 | KX022775 | KX022729 | North Tarim /Weigan River | 7 |
| *D.maculatus* | W15 | R1 | 1305241 | KX022709 | KX022740 | KX022775 | KX022729 | North Tarim /Weigan River | 7 |
| *D.maculatus* | W16 | R1 | 1305236 | KX022704 | KX022745 | KX022777 | KX022729 | North Tarim /Weigan River | 7 |
| *D.maculatus* | W16 | R1 | 1305243 | KX022704 | KX022745 | KX022777 | KX022729 | North Tarim /Weigan River | 7 |
| *D.maculatus* | W17 | R1 | 1305245 | KX022704 | KX022745 | KX022780 | KX022729 | North Tarim /Weigan River | 7 |
| *D.maculatus* | A1 | R2 | 1305258 | KJ081424 | KX022746 | KX022781 | KX022730 | North Tarim /Aksu River | 9 |
| *D.maculatus* | A1 | R2 | 1305259 | KJ081424 | KX022746 | KX022781 | KX022730 | North Tarim /Aksu River | 9 |
| *D.maculatus* | A1 | R2 | 1305261 | KJ081424 | KX022746 | KX022781 | KX022730 | North Tarim /Aksu River | 9 |
| *D.maculatus* | A1 | R2 | 1305264 | KJ081424 | KX022746 | KX022781 | KX022730 | North Tarim /Aksu River | 9 |
| *D.maculatus* | A1 | R2 | 1305266 | KJ081424 | KX022746 | KX022781 | KX022730 | North Tarim /Aksu River | 9 |
| *D.maculatus* | A1 | R2 | 1305272 | KJ081424 | KX022746 | KX022781 | KX022730 | North Tarim /Aksu River | 9 |
| *D.maculatus* | A1 | R2 | 1305276 | KJ081424 | KX022746 | KX022781 | KX022730 | North Tarim /Aksu River | 8 |
| *D.maculatus* | A1 | R2 | 1305277 | KJ081424 | KX022746 | KX022781 | KX022730 | North Tarim /Aksu River | 8 |
| *D.maculatus* | A1 | R2 | 1305278 | KJ081424 | KX022746 | KX022781 | KX022730 | North Tarim /Aksu River | 8 |
| *D.maculatus* | A1 | R2 | 1305280 | KJ081424 | KX022746 | KX022781 | KX022730 | North Tarim /Aksu River | 8 |
| *D.maculatus* | A2 | R2 | 1305260 | KJ081424 | KX022747 | KX022781 | KX022730 | North Tarim /Aksu River | 9 |
| *D.maculatus* | A2 | R2 | 1305267 | KJ081424 | KX022747 | KX022781 | KX022730 | North Tarim /Aksu River | 9 |
| *D.maculatus* | A2 | R2 | 1305274 | KJ081424 | KX022747 | KX022781 | KX022730 | North Tarim /Aksu River | 8 |
| *D.maculatus* | A2 | R2 | 1305279 | KJ081424 | KX022747 | KX022781 | KX022730 | North Tarim /Aksu River | 8 |
| *D.maculatus* | A3 | R2 | 1305262 | KX022710 | KX022748 | KX022782 | KX022730 | North Tarim /Aksu River | 9 |
| *D.maculatus* | A4 | R2 | 1305263 | KJ081424 | KX022746 | KX022783 | KX022730 | North Tarim /Aksu River | 9 |
| *D.maculatus* | A4 | R2 | 1305265 | KJ081424 | KX022746 | KX022783 | KX022730 | North Tarim /Aksu River | 9 |
| *D.maculatus* | A4 | R2 | 1305268 | KJ081424 | KX022746 | KX022783 | KX022730 | North Tarim /Aksu River | 9 |
| *D.maculatus* | A4 | R2 | 1305270 | KJ081424 | KX022746 | KX022783 | KX022730 | North Tarim /Aksu River | 9 |
| *D.maculatus* | A5 | R2 | 1305269 | KJ081424 | KX022749 | KX022781 | KX022730 | North Tarim /Aksu River | 9 |
| *D.maculatus* | A6 | R2 | 1305271 | KJ081424 | KX022750 | KX022781 | KX022730 | North Tarim /Aksu River | 9 |
| *D.maculatus* | A7 | R2 | 1305273 | KX022711 | KX022746 | KX022783 | KX022730 | North Tarim /Aksu River | 9 |
| *D.maculatus* | A8 | R2 | 1305275 | KX022712 | KX022748 | KX022784 | KX022730 | North Tarim /Aksu River | 8 |
| *D.maculatus* | K1 | R2 | 1305281 | KX022713 | KX022751 | KX022785 | KX022730 | North Tarim /Kashgar River | 10 |
| *D.maculatus* | K2 | R3 | 1305282 | KX022714 | KX022751 | KX022786 | KX022731 | North Tarim /Kashgar River | 11 |
| *D.maculatus* | K3 | R3 | 1305283 | KX022715 | KX022752 | KX022787 | KX022731 | North Tarim /Kashgar River | 11 |
| *D.maculatus* | K4 | R3 | 1305284 | KX022716 | KX022753 | KX022786 | KX022731 | North Tarim /Kashgar River | 11 |
| *D.maculatus* | Y1 | R4 | 1007135 | KX022688 | KX022733 | KX022759 | KX022726 | South Tarim / Yarkand River | 13 |
| *D.maculatus* | Y1 | R4 | 1007137 | KX022688 | KX022733 | KX022759 | KX022726 | South Tarim / Yarkand River | 13 |
| *D.maculatus* | Y1 | R4 | 1007115 | KX022688 | KX022733 | KX022759 | KX022726 | South Tarim / Yarkand River | 13 |
| *D.maculatus* | Y1 | R4 | 1007120 | KX022688 | KX022733 | KX022759 | KX022726 | South Tarim / Yarkand River | 13 |
| *D.maculatus* | Y1 | R4 | 1007122 | KX022688 | KX022733 | KX022759 | KX022726 | South Tarim / Yarkand River | 13 |
| *D.maculatus* | Y1 | R4 | 1007124 | KX022688 | KX022733 | KX022759 | KX022726 | South Tarim / Yarkand River | 13 |
| *D.maculatus* | Y1 | R4 | 1007125 | KX022688 | KX022733 | KX022759 | KX022726 | South Tarim / Yarkand River | 13 |
| *D.maculatus* | Y1 | R4 | 1007126 | KX022688 | KX022733 | KX022759 | KX022726 | South Tarim / Yarkand River | 13 |
| *D.maculatus* | Y1 | R4 | 1007157 | KX022688 | KX022733 | KX022759 | KX022726 | South Tarim / Yarkand River | 14 |
| *D.maculatus* | Y1 | R4 | 1007158 | KX022688 | KX022733 | KX022759 | KX022726 | South Tarim / Yarkand River | 14 |
| *D.maculatus* | Y1 | R4 | 1007159 | KX022688 | KX022733 | KX022759 | KX022726 | South Tarim / Yarkand River | 14 |
| *D.maculatus* | Y1 | R4 | 1007160 | KX022688 | KX022733 | KX022759 | KX022726 | South Tarim / Yarkand River | 14 |
| *D.maculatus* | Y1 | R4 | 1007161 | KX022688 | KX022733 | KX022759 | KX022726 | South Tarim / Yarkand River | 14 |
| *D.maculatus* | Y1 | R4 | 1007165 | KX022688 | KX022733 | KX022759 | KX022726 | South Tarim / Yarkand River | 14 |
| *D.maculatus* | Y1 | R4 | 1007167 | KX022688 | KX022733 | KX022759 | KX022726 | South Tarim / Yarkand River | 14 |
| *D.maculatus* | Y1 | R4 | 1007168 | KX022688 | KX022733 | KX022759 | KX022726 | South Tarim / Yarkand River | 14 |
| *D.maculatus* | Y1 | R4 | 1007128 | KX022688 | KX022733 | KX022759 | KX022726 | South Tarim / Yarkand River | 13 |
| *D.maculatus* | Y1 | R4 | 1007133 | KX022688 | KX022733 | KX022759 | KX022726 | South Tarim / Yarkand River | 13 |
| *D.maculatus* | Y1 | R4 | 1007169 | KX022688 | KX022733 | KX022759 | KX022726 | South Tarim / Yarkand River | 14 |
| *D.maculatus* | Y1 | R4 | 1007171 | KX022688 | KX022733 | KX022759 | KX022726 | South Tarim / Yarkand River | 14 |
| *D.maculatus* | Y1 | R4 | 1007172 | KX022688 | KX022733 | KX022759 | KX022726 | South Tarim / Yarkand River | 14 |
| *D.maculatus* | Y1 | R4 | 1007173 | KX022688 | KX022733 | KX022759 | KX022726 | South Tarim / Yarkand River | 14 |
| *D.maculatus* | Y2 | R4 | 1007136 | KX022689 | KX022733 | KX022760 | KX022726 | South Tarim / Yarkand River | 13 |
| *D.maculatus* | Y2 | R4 | 1007132 | KX022689 | KX022733 | KX022760 | KX022726 | South Tarim / Yarkand River | 13 |
| *D.maculatus* | Y3 | R4 | 1007138 | KX022690 | KX022733 | KX022759 | KX022726 | South Tarim / Yarkand River | 13 |
| *D.maculatus* | Y4 | R4 | 1007139 | KX022691 | KX022733 | KX022761 | KX022726 | South Tarim / Yarkand River | 13 |
| *D.maculatus* | Y5 | R4 | 1007140 | KX022692 | KX022733 | KX022762 | KX022726 | South Tarim / Yarkand River | 13 |
| *D.maculatus* | Y5 | R4 | 1007148 | KX022692 | KX022733 | KX022762 | KX022726 | South Tarim / Yarkand River | 13 |
| *D.maculatus* | Y5 | R4 | 1007116 | KX022692 | KX022733 | KX022762 | KX022726 | South Tarim / Yarkand River | 13 |
| *D.maculatus* | Y5 | R4 | 1007127 | KX022692 | KX022733 | KX022762 | KX022726 | South Tarim / Yarkand River | 13 |
| *D.maculatus* | Y6 | R4 | 1007141 | KX022693 | KX022733 | KX022763 | KX022726 | South Tarim / Yarkand River | 13 |
| *D.maculatus* | Y7 | R4 | 1007142 | KX022694 | KX022733 | KX022760 | KX022726 | South Tarim / Yarkand River | 13 |
| *D.maculatus* | Y7 | R4 | 1007147 | KX022694 | KX022733 | KX022760 | KX022726 | South Tarim / Yarkand River | 13 |
| *D.maculatus* | Y8 | R4 | 1007143 | KX022688 | KX022733 | KX022760 | KX022726 | South Tarim / Yarkand River | 13 |
| *D.maculatus* | Y8 | R4 | 1007163 | KX022688 | KX022733 | KX022760 | KX022726 | South Tarim / Yarkand River | 14 |
| *D.maculatus* | Y8 | R5 | 1007164 | KX022688 | KX022733 | KX022760 | KX022727 | South Tarim / Yarkand River | 14 |
| *D.maculatus* | Y8 | R4 | 1007166 | KX022688 | KX022733 | KX022760 | KX022726 | South Tarim / Yarkand River | 14 |
| *D.maculatus* | Y8 | R5 | 1007129 | KX022688 | KX022733 | KX022760 | KX022727 | South Tarim / Yarkand River | 13 |
| *D.maculatus* | Y8 | R4 | 1007131 | KX022688 | KX022733 | KX022760 | KX022726 | South Tarim / Yarkand River | 13 |
| *D.maculatus* | Y9 | R4 | 1007144 | KX022692 | KX022734 | KX022764 | KX022726 | South Tarim / Yarkand River | 13 |
| *D.maculatus* | Y10 | R4 | 1007145 | KX022695 | KX022733 | KX022760 | KX022726 | South Tarim / Yarkand River | 13 |
| *D.maculatus* | Y11 | R4 | 1007146 | KX022696 | KX022733 | KX022765 | KX022726 | South Tarim / Yarkand River | 13 |
| *D.maculatus* | Y12 | R4 | 1305285 | KX022692 | KX022733 | KX022764 | KX022726 | South Tarim / Yarkand River | 12 |
| *D.maculatus* | Y12 | R4 | 1305287 | KX022692 | KX022733 | KX022764 | KX022726 | South Tarim / Yarkand River | 12 |
| *D.maculatus* | Y12 | R5 | 1305288 | KX022692 | KX022733 | KX022764 | KX022727 | South Tarim / Yarkand River | 12 |
| *D.maculatus* | Y12 | R4 | 1305289 | KX022692 | KX022733 | KX022764 | KX022726 | South Tarim / Yarkand River | 12 |
| *D.maculatus* | Y13 | R4 | 1305286 | KX022692 | KX022733 | KX022788 | KX022726 | South Tarim / Yarkand River | 12 |
| *D.maculatus* | Y14 | R4 | 1305290 | KX022717 | KX022733 | KX022764 | KX022726 | South Tarim / Yarkand River | 12 |
| *D.maculatus* | Y15 | R4 | 1007117 | KX022692 | KX022757 | KX022762 | KX022726 | South Tarim / Yarkand River | 13 |
| *D.maculatus* | Y16 | R5 | 1007118 | KX022720 | KX022758 | KX022759 | KX022727 | South Tarim / Yarkand River | 13 |
| *D.maculatus* | Y16 | R5 | 1007134 | KX022720 | KX022758 | KX022759 | KX022727 | South Tarim / Yarkand River | 13 |
| *D.maculatus* | Y17 | R4 | 1007119 | KX022721 | KX022733 | KX022762 | KX022726 | South Tarim / Yarkand River | 13 |
| *D.maculatus* | Y18 | R4 | 1007121 | KX022722 | KX022734 | KX022764 | KX022726 | South Tarim / Yarkand River | 13 |
| *D.maculatus* | Y19 | R4 | 1007123 | KX022696 | KX022733 | KX022759 | KX022726 | South Tarim / Yarkand River | 13 |
| *D.maculatus* | Y20 | R4 | 1007162 | KX022725 | KX022733 | KX022760 | KX022726 | South Tarim / Yarkand River | 14 |
| *D.maculatus* | Y21 | R4 | 1007130 | KX022693 | KX022733 | KX022762 | KX022726 | South Tarim / Yarkand River | 13 |
| *D.maculatus* | Y22 | R4 | 1007170 | KX022720 | KX022758 | KX022760 | KX022726 | South Tarim / Yarkand River | 14 |
| *D.maculatus* | H1 | R5 | 1007154 | KX022697 | KX022735 | KX022766 | KX022727 | South Tarim /Hotan River | 16 |
| *D.maculatus* | H1 | R5 | 1007155 | KX022697 | KX022735 | KX022766 | KX022727 | South Tarim /Hotan River | 16 |
| *D.maculatus* | H1 | R5 | 1007149 | KX022697 | KX022735 | KX022766 | KX022727 | South Tarim /Hotan River | 15 |
| *D.maculatus* | H1 | R5 | 1007152 | KX022697 | KX022735 | KX022766 | KX022727 | South Tarim /Hotan River | 15 |
| *D.maculatus* | H2 | R5 | 1007156 | KX022698 | KX022735 | KX022766 | KX022727 | South Tarim /Hotan River | 16 |
| *D.maculatus* | H2 | R5 | 1007150 | KX022698 | KX022735 | KX022766 | KX022727 | South Tarim /Hotan River | 15 |
| *D.maculatus* | H3 | R5 | 1007151 | KX022723 | KX022735 | KX022766 | KX022727 | South Tarim /Hotan River | 15 |
| *D.maculatus* | H4 | R5 | 1007153 | KX022724 | KX022735 | KX022766 | KX022727 | South Tarim /Hotan River | 16 |
| *D.maculatus* | IN1 | R7 | 1407941 | KX022718 | KX022754 | KX022789 | KX022732 | Indus River | 4 |
| *D.maculatus* | IN1 | R7 | 1407942 | KX022718 | KX022754 | KX022789 | KX022732 | Indus River | 4 |
| *D.maculatus* | IN1 | R7 | 1407943 | KX022718 | KX022754 | KX022789 | KX022732 | Indus River | 4 |
| *D.maculatus* | IN1 | R7 | 1407944 | KX022718 | KX022754 | KX022789 | KX022732 | Indus River | 4 |
| *D.maculatus* | IN1 | R7 | 1407945 | KX022718 | KX022754 | KX022789 | KX022732 | Indus River | 4 |
| *D.maculatus* | IN1 | R7 | 1407950 | KX022718 | KX022754 | KX022789 | KX022732 | Indus River | 4 |
| *D.maculatus* | IN1 | R7 | 1407951 | KX022718 | KX022754 | KX022789 | KX022732 | Indus River | 4 |
| *D.maculatus* | IN1 | R7 | 1407953 | KX022718 | KX022754 | KX022789 | KX022732 | Indus River | 4 |
| *D.maculatus* | IN1 | R7 | 1407956 | KX022718 | KX022754 | KX022789 | KX022732 | Indus River | 4 |
| *D.maculatus* | IN1 | R7 | 1407957 | KX022718 | KX022754 | KX022789 | KX022732 | Indus River | 4 |
| *D.maculatus* | IN1 | R7 | 1407960 | KX022718 | KX022754 | KX022789 | KX022732 | Indus River | 4 |
| *D.maculatus* | IN1 | R7 | 1407961 | KX022718 | KX022754 | KX022789 | KX022732 | Indus River | 4 |
| *D.maculatus* | IN1 | R7 | 1407962 | KX022718 | KX022754 | KX022789 | KX022732 | Indus River | 4 |
| *D.maculatus* | IN1 | R7 | 1407967 | KX022718 | KX022754 | KX022789 | KX022732 | Indus River | 5 |
| *D.maculatus* | IN1 | R7 | 1407970 | KX022718 | KX022754 | KX022789 | KX022732 | Indus River | 5 |
| *D.maculatus* | IN1 | R7 | 1407971 | KX022718 | KX022754 | KX022789 | KX022732 | Indus River | 5 |
| *D.maculatus* | IN1 | R7 | 1407973 | KX022718 | KX022754 | KX022789 | KX022732 | Indus River | 5 |
| *D.maculatus* | IN1 | R7 | 1407974 | KX022718 | KX022754 | KX022789 | KX022732 | Indus River | 5 |
| *D.maculatus* | IN2 | R7 | 1407947 | KX022719 | KX022755 | KX022790 | KX022732 | Indus River | 4 |
| *D.maculatus* | IN2 | R7 | 1407952 | KX022719 | KX022755 | KX022790 | KX022732 | Indus River | 4 |
| *D.maculatus* | IN2 | R7 | 1407959 | KX022719 | KX022755 | KX022790 | KX022732 | Indus River | 4 |
| *D.maculatus* | IN2 | R7 | 1407964 | KX022719 | KX022755 | KX022790 | KX022732 | Indus River | 4 |
| *D.maculatus* | IN2 | R7 | 1407965 | KX022719 | KX022755 | KX022790 | KX022732 | Indus River | 4 |
| *D.maculatus* | IN2 | R7 | 1407969 | KX022719 | KX022755 | KX022790 | KX022732 | Indus River | 5 |
| *D.maculatus* | IN2 | R7 | 1407972 | KX022719 | KX022755 | KX022790 | KX022732 | Indus River | 5 |
| *D.maculatus* | IN2 | R7 | 1407975 | KX022719 | KX022755 | KX022790 | KX022732 | Indus River | 5 |
| *D.maculatus* | IN3 | R7 | 1407948 | KX022719 | KX022756 | KX022791 | KX022732 | Indus River | 4 |
| *D.maculatus* | IN3 | R7 | 1407954 | KX022719 | KX022756 | KX022791 | KX022732 | Indus River | 4 |
| *D.maculatus* | IN3 | R7 | 1407955 | KX022719 | KX022756 | KX022791 | KX022732 | Indus River | 4 |
| *D.maculatus* | IN3 | R7 | 1407958 | KX022719 | KX022756 | KX022791 | KX022732 | Indus River | 4 |
| *D.maculatus* | IN3 | R7 | 1407963 | KX022719 | KX022756 | KX022791 | KX022732 | Indus River | 4 |
| *D.maculatus* | IN3 | R7 | 1407966 | KX022719 | KX022756 | KX022791 | KX022732 | Indus River | 4 |
| *D.maculatus* | IN3 | R7 | 1407976 | KX022719 | KX022756 | KX022791 | KX022732 | Indus River | 5 |
| *D.maculatus* | IN3 | R7 | 1407977 | KX022719 | KX022756 | KX022791 | KX022732 | Indus River | 5 |
| Outgroup |  |  |  |  |  |  |  |  |  |
| *G. dybowskii* |  |  |  | KJ081423 | KJ081377 |  |  |  |  |
| *A. laticeps* |  |  |  | KF564793 | KF564793 | KF564793 |  |  |  |
| *B. barbus* |  |  |  | AB238965 | AB238965 | AB238965 |  |  |  |
