# Supplementary material for: Phylogeography of Diptychus maculatus (Cyprinidae) endemic to the northern margin of the QTP and Tien Shan region
Source: BMC Evol Biol. 2016 Sep 9;16(1):186. doi: 10.1186/s12862-016-0756-3 (PMC5017051; doi:10.1186/s12862-016-0756-3)
Supplement: Additional file 3: Table S2. — The F ST values among the populations of Diptychus maculatus based on the combined mtDNA data. (DOCX 21 kb) [file 12862_2016_756_MOESM3_ESM.docx]

**Table S2** The *F*_ST_ values among the populations of *Diptychus maculatus* based on the combined mtDNA data.

| Lineage |  | Ili | River |  |  | Indus | River |  | North | Tarim | River |  |  |  |  | South | Tarim | River |  |
| --- | --- | --- | --- | --- | --- | --- | --- | --- | --- | --- | --- | --- | --- | --- | --- | --- | --- | --- | --- |
|  | Code | 1 | 2 | 3 |  | 4 | 5 |  | 6 | 7 | 8 | 9 | 10 | 11 |  | 12 | 13 | 14 | 15 |
|  |  |  |  |  |  |  |  |  |  |  |  |  |  |  |  |  |  |  |  |
| Ili | 2 | 0.492** |  |  |  |  |  |  |  |  |  |  |  |  |  |  |  |  |  |
|  | 3 | 0.448** | 0.387** |  |  |  |  |  |  |  |  |  |  |  |  |  |  |  |  |
| Indus | 4 | 0.960** | 0.970** | 0.963** |  |  |  |  |  |  |  |  |  |  |  |  |  |  |  |
|  | 5 | 0.961** | 0.974** | 0.966** |  | -0.069 |  |  |  |  |  |  |  |  |  |  |  |  |  |
| North | 6 | 0.968** | 0.974** | 0.971** |  | 0.974** | 0.974** |  |  |  |  |  |  |  |  |  |  |  |  |
| Tarim | 7 | 0.960** | 0.967** | 0.961** |  | 0.968** | 0.967** |  | 0.017 |  |  |  |  |  |  |  |  |  |  |
|  | 8 | 0.959** | 0.973** | 0.962** |  | 0.963** | 0.959** |  | 0.967** | 0.957** |  |  |  |  |  |  |  |  |  |
|  | 9 | 0.966** | 0.976** | 0.970** |  | 0.968** | 0.969** |  | 0.970** | 0.962** | -0.051 |  |  |  |  |  |  |  |  |
|  | 10 | 0.970 | 0.984 | 0.982 |  | 0.969 | 0.967 |  | 0.968 | 0.957 | 0.924 | 0.956 |  |  |  |  |  |  |  |
|  | 11 | 0.968** | 0.981** | 0.975** |  | 0.968** | 0.966** |  | 0.967** | 0.956** | 0.926** | 0.952** | 0.090 |  |  |  |  |  |  |
| South | 12 | 0.972** | 0.984 | 0.984** |  | 0.971** | 0.974** |  | 0.975** | 0.967** | 0.972** | 0.978** | 0.993 | 0.984* |  |  |  |  |  |
| Tarim | 13 | 0.908** | 0.923** | 0.901** |  | 0.930** | 0.918** |  | 0.945** | 0.939** | 0.912** | 0.926** | 0.914* | 0.917** |  | 0.466** |  |  |  |
|  | 14 | 0.976** | 0.983 | 0.982** |  | 0.975** | 0.978** |  | 0.978** | 0.971** | 0.977** | 0.980** | 0.987 | 0.985** |  | 0.929** | 0.168** |  |  |
|  | 15 | 0.971** | 0.983 | 0.981** |  | 0.970** | 0.971** |  | 0.977** | 0.969** | 0.960** | 0.971** | 0.986 | 0.978* |  | 0.986** | 0.892** | 0.982** |  |
|  | 16 | 0.971** | 0.984 | 0.983** |  | 0.969** | 0.969** |  | 0.977** | 0.968** | 0.957* | 0.971** | 0.992 | 0.978 |  | 0.990* | 0.888** | 0.983** | -0.207 |

Significant pairwise differences: **P* < 0.05; ***P* < 0.01. Locality codes correspond to those in Fig.1 and Table S1.
