# Supplementary material for: Phylogeography of Diptychus maculatus (Cyprinidae) endemic to the northern margin of the QTP and Tien Shan region
Source: BMC Evol Biol. 2016 Sep 9;16(1):186. doi: 10.1186/s12862-016-0756-3 (PMC5017051; doi:10.1186/s12862-016-0756-3)
Supplement: Additional file 4: Table S3. — Mean divergence values between clades for Diptychus maculatus. (DOCX 16 kb) [file 12862_2016_756_MOESM4_ESM.docx]

**Table S3** Mean divergence values between clades for *Diptychus maculatus*.

|  | North Tarim | South Tarim | Ili River | Indus River |
| --- | --- | --- | --- | --- |
| North Tarim |  | ** | ** | ** |
| South Tarim | 0.03011 |  | ** | ** |
| Ili River | 0.02401 | 0.02329 |  | ** |
| Indus River | 0.03081 | 0.02936 | 0.02259 |  |

Distance values are below diagonal, and *P* values are above diagonal, where ‘**’ indicates *P* < 0.01.
